# The effectiveness, implementation, and experiences of peer support approaches for mental health: a systematic umbrella review

**DOI:** 10.1186/s12916-024-03260-y

**Published:** 2024-02-29

**Authors:** Ruth E. Cooper, Katherine R. K. Saunders, Anna Greenburgh, Prisha Shah, Rebecca Appleton, Karen Machin, Tamar Jeynes, Phoebe Barnett, Sophie M. Allan, Jessica Griffiths, Ruth Stuart, Lizzie Mitchell, Beverley Chipp, Stephen Jeffreys, Brynmor Lloyd-Evans, Alan Simpson, Sonia Johnson

**Affiliations:** 1 NIHR Mental Health Policy Research Unit, Institute of Psychiatry, Psychology and Neuroscience, King’s College London, London, UK; 2 NIHR Mental Health Policy Research Unit, Division of Psychiatry, University College London, London, UK; 3 Centre for Outcomes Research and Effectiveness, Research Department of Clinical, Educational and Health Psychology, University College London, London, UK; 4 National Collaborating Centre for Mental Health, Royal College of Psychiatrists, London, UK; 5 University of East Anglia, Norwich, UK; 6 MHPRU Lived Experience Working Group, London, UK; 7 Florence Nightingale Faculty of Nursing, Midwifery and Palliative Care, London, UK; 8 Camden and Islington NHS Foundation Trust, London, UK

**Keywords:** Peer support, Mental health, Systematic review, Umbrella review

## Abstract

**Background:**

Peer support for mental health is recommended across international policy guidance and provision. Our systematic umbrella review summarises evidence on the effectiveness, implementation, and experiences of paid peer support approaches for mental health.

**Methods:**

We searched MEDLINE, EMBASE, PsycINFO, The Campbell Collaboration, and The Cochrane Database of Systematic Reviews (2012–2022) for reviews of paid peer support interventions for mental health. The AMSTAR2 assessed quality. Results were synthesised narratively, with implementation reported using the CFIR (Consolidated Framework for Implementation Research). The protocol was registered with PROSPERO (registration number: CRD42022362099).

**Results:**

We included 35 reviews (426 primary studies, *n* = 95–40,927 participants): systematic reviews with (*n* = 13) or without (*n* = 13) meta-analysis, or with qualitative synthesis (*n* = 3), scoping reviews (*n* = 6). Most reviews were low or critically low (97%) quality, one review was high quality. Effectiveness was investigated in 23 reviews. Results were mixed; there was some evidence from meta-analyses that peer support may improve depression symptoms (particularly perinatal depression), self-efficacy, and recovery. Factors promoting successful implementation, investigated in 9 reviews, included adequate training and supervision, a recovery-oriented workplace, strong leadership, and a supportive and trusting workplace culture with effective collaboration. Barriers included lack of time, resources and funding, and lack of recognised peer support worker (PSW) certification. Experiences of peer support were explored in 11 reviews, with 3 overarching themes: (i) what the PSW role can bring, including recovery and improved wellbeing for service users and PSWs; (ii) confusion over the PSW role, including role ambiguity and unclear boundaries; and (iii) organisational challenges and impact, including low pay, negative non-peer staff attitudes, and lack of support and training.

**Conclusions:**

Peer support may be effective at improving some clinical outcomes, self-efficacy, and recovery. Certain populations, e.g. perinatal populations, may especially benefit from peer support. Potential strategies to successfully implement PSWs include co-production, clearly defined PSW roles, a receptive hierarchical structure and staff, appropriate PSW and staff training with clinical and/or peer supervision alongside safeguarding. Services could benefit from clear, coproduced, setting specific implementation guidelines for PSW. PSW roles tend to be poorly defined and associations between PSW intervention content and impacts need further investigation. Future research should reflect the priorities of providers/service users involved in peer support.

**Supplementary Information:**

The online version contains supplementary material available at 10.1186/s12916-024-03260-y.

## Background

Peer support in mental health care is a recovery-orientated approach delivered by individuals who have lived experience of mental health difficulties (as service users, carers, parents or supporters). Peer support workers (PSWs) are employed to draw on these experiences to support mental health service users or carers of people with mental health conditions [1, 2]. As such, PSWs are uniquely positioned to facilitate recovery through empathic engagement with service users and their support networks. The success of peer support is thought to be based in the sharing of lived experiences and mental health knowledge and through interpersonal connection [3, 4]. Across diagnoses, peer support may promote recovery through the modelling of coping strategies, and by providing hope and an example of recovery to those dealing with mental health difficulties [5].

Peer support has been utilised across various populations and types of service, for example in services for early intervention in psychosis [6], for people with co-occurring substance abuse and mental health difficulties [7], and in community interventions to reduce mental health inpatient admissions [8]. The format of peer support varies across services, for example it may involve one-to-one or group sessions, online or face-to-face delivery, unstructured open-ended conversations or more structured manualised support, or activities such as walking groups [9, 10]. Peer support may be delivered by trained peer support staff or on a more ad hoc basis among peers [11]. Peer support for mental health takes place within mental health services in both statutory and voluntary sector settings [11]. Although PSWs may be paid or unpaid [6, 12], paid roles have become increasingly available in mental health care settings [13]. Professionalising PSW roles as paid demonstrates the value of the role and appropriately rewards work done, should ensure formal training, supervision and management, and may help to clarify the boundaries of the role [14].

Service user networks and researchers in relevant fields have strongly advocated for provision of peer support [14, 15], and peer support is now recognised and recommended across international mental health policy guidance, reflecting an increased understanding of the value of embedding lived experience support in formal mental health services [16–20]. In the UK, peer support is currently being expanded in the NHS [16].

There have been many reviews of the peer support literature separately evaluating the efficacy, implementation, and experiences of peer support from a variety of different perspectives (e.g. [21–24]). Given the numerous and sometimes inconclusive results from existing reviews on this topic, our research group, the NIHR Mental Health Policy Research Unit, agreed with policy makers in England to conduct an umbrella review of peer support to provide clinicians, policy makers and researchers with an overall assessment on the evidence available, comparing results between reviews, while taking the quality of these reviews into account [25, 26]. The aim of this systematic umbrella review is to collate, synthesise and summarise the available evidence from published reviews to address the following research questions:

1. What is the effectiveness (e.g. clinical, social, functional) and cost-effectiveness of paid peer support approaches for mental health?
2. What influences the implementation of peer support approaches for mental health?
3. What are the experiences of peer support approaches for mental health (e.g. of acceptability) from the perspective of PSWs, healthcare practitioners, service users, carers?

## Methods

This umbrella review was conducted by the NIHR Mental Health Policy Research Unit (MHPRU), based at King’s College London and University College London, which delivers evidence to inform government and NHS policy in England, agreeing a programme of rapid research with policymakers.

### Study design and protocol

We conducted a systematic umbrella review following guidance from Fusar-Poli et al. [27] and Cochrane [28]. The review is reported according to the Preferred Reporting Items for Systematic Reviews and Meta-Analyses (PRISMA) (see Additional file 1: Appendix 1 for the PRISMA checklist) [29]. The protocol was registered with PROSPERO (registration number: CRD42022362099) [30]. One amendment was made to the protocol after registration. We amended the ‘intervention’ section to state that reviews were excluded if the majority of interventions did not meet eligibility criteria, e.g. because we found that reviews often included paid and unpaid peer support interventions and did not report results separately.

### Lived experience researcher involvement

Members of the MHPRU Lived Experience Working Group (LEWG), who collectively have substantial experience of delivering or receiving peer support, contributed extensively to this review, including protocol development, study selection, data extraction, quality appraisal, data synthesis, drafting the manuscript and lived experience commentary, and attending working group meetings.

### Eligibility criteria

The eligibility criteria are detailed in full in the protocol [30]. In summary, we included:

- *Study designs*: Published, peer-reviewed systematic, scoping or realist reviews which synthesised quantitative or qualitative data (narratively or formally using, e.g. a meta-analysis or meta-synthesis) that examined outcomes or experiences relevant to our research questions.
- *Intervention*: We defined peer support as ‘involving a person who has lived experience of mental health condition(s), or caring for those with mental health conditions, being employed to use and draw on their experiences and empathy to support service users who have mental health conditions or carers or parents of people with mental health conditions.’ Eligible peer support approaches were paid, meaning that the PSW was paid for their work, and delivered face-to-face or remotely, for people with mental health conditions or for carers of people with mental health conditions, across any mental healthcare settings. Peer support approaches were ineligible if the PSWs were not in a dedicated peer support role, if they were primarily for physical health, or automated (i.e. peer support ‘bots’ or avatars). We excluded reviews where over 50% of primary studies in the review did not meet eligibility criteria, e.g. if the majority of people delivering the interventions were unpaid.
- *Population*: Children, young people and adults with a mental health condition (including substance use disorders), carers, paid PSWs and mental healthcare practitioners working alongside PSWs. We excluded service users with a primary diagnosis of an organic mental disorder (e.g. dementia), neurodevelopmental disorders, acquired cognitive impairment and adjustment disorders.
- *Outcome measures*: Included reviews reported outcomes or data on at least one of the following peer support related outcomes that addressed our research questions: (i) clinical outcomes, (ii) economic or cost-effectiveness, (iii) recovery outcomes, e.g. hope, empowerment, goal-attainment, quality of life, (iv) social outcomes, (v) implementation outcomes and barriers and facilitators to implementation, (vi) experiences of delivering, receiving or working alongside peer support and (vii) theories of what works for whom in peer support.

### Information sources and search strategy

We combined terms for peer support, reviews and mental health conditions using Boolean operators (AND, OR). We searched the following databases: MEDLINE, EMBASE, PsycINFO, The Campbell Collaboration and The Cochrane Database of Systematic Reviews (see Additional file 1: Appendix 2 for full search strategy). Searches were run from January 2012 to November 2022 as these reviews will include primary research published before 2012 [31]. There was no time limit for the primary papers in the included reviews. We had no language restrictions.

### Selection process

Reviewers (KS, RC, JG, RS, RA, KM, PS, SA) screened titles and abstracts, and subsequently full texts. To ensure consistent application of eligibility criteria all reviewers initially independently screened the same ten titles and abstracts and discussed inclusion/exclusion. The remaining titles and abstracts were then screened. Records were double screened blind by two reviewers at both the title and abstract (94% agreement) and full text (86% agreement) stages. All disagreements were resolved through discussion with the study team.

### Data extraction

Data extraction was completed in Microsoft Excel by the review team (RC, KS, KM, PS, JG, RS, PB, RA). The data used in the paper were checked by another member of the review team. The extracted data included basic information about reviews (e.g. number of included studies, number of participants, review type, aim/objectives), basic information about primary studies (e.g. references, designs), search strategy (e.g. databases searched, eligibility criteria), population (e.g. gender, age), peer support approach (e.g. peer support type and description), type of comparator, additional information (e.g. quality appraisal methods, review author conclusions), primary and secondary outcomes of systematic review or qualitative results.

### Quality appraisal of included reviews

The quality of included reviews was independently assessed by reviewers (RC, KS, KM, PS, JG, RS, PB, RA) using the AMSTAR 2 (A MeaSurement Tool to Assess systematic Reviews), a 16-point tool for assessment of the methodological quality of systematic reviews [32]. We adapted the AMSTAR 2 to apply for scoping reviews and systematic reviews of qualitative data (described in full in Additional file 1: Appendix 3). The following questions were adapted: (1) PICO criteria, (2) Protocol requirements, (8) Detail of included studies, (9) Risk of Bias requirement. Two reviewers (KS, AG) 100% double-scored reviews blind with any outstanding disagreements resolved through discussion between AG, KS, and RC. Overall ratings for each study were calculated according to guidance [32], based on 7 critical domains and 6 non-critical domains within the AMSTAR 2 tool. Studies with no or one non-critical weakness and no critical flaws were rated as high quality. Studies with more than one non-critical weakness and no critical weaknesses were rated as moderate quality. Studies with one critical flaw irrespective of non-critical weaknesses were rated as low quality, and those with more than one critical flaw irrespective of non-critical weaknesses were rated as critically low quality. The AMSTAR 2 guidance [32] states that reviews of critically low quality should not be relied on for comprehensive and accurate summaries of the literature.

### Synthesis methods

#### RQ 1: What is the effectiveness (e.g. clinical, social, functional) and cost-effectiveness of paid peer support approaches for mental health?

Data were tabulated and summarised narratively by two researchers (KS, AG); effectiveness meta-analysis data calculated from two or more studies were tabulated separately from non-meta-analysis effectiveness outcomes. Review outcomes were similar, but not similar enough to combine meaningfully in a meta-analysis. Effect sizes (with 95% CIs and *p*-values) were reported along with *I*^2^ statistic (with 95% CIs, *p*-values, *χ*^2^, and degrees of freedom) where available. We did not tabulate data for subgroup analyses.

#### RQ 2: What influences the implementation of peer support approaches for mental health?

Outcomes were tabulated according to the main domains in the Consolidated Framework for Implementation Research (CFIR) [33]. The CFIR provides a comprehensive framework, composed of 5 domains, associated with the effective implementation of interventions [33]. The 5 domains are as follows: Innovation (the ‘thing’ being implemented); Outer setting (the setting in which the inner setting exists, e.g. hospital system); Inner setting (the setting in which the innovation is implemented, e.g. hospital); Individuals (the roles and characteristics of individuals); Implementation process (the activities and strategies used to implement the innovation) [33]. Synthesis was conducted using a collaborative process involving one member of the study team (RA) and one lived experience researcher (PS).

#### RQ 3: What are the experiences of peer support approaches for mental health (e.g. of acceptability) from the perspective of PSWs, healthcare practitioners, service users and carers?

Experiences were synthesised narratively, by three researchers, including two lived experience researchers (TJ, KM, RC) [34]. Themes from reviews which were identified as addressing research question 3 were extracted and similar themes across the reviews were grouped together. Each group was accounted for using an existing theme from one or more of the reviews or if this was not possible a new theme was developed. Three overarching themes were identified through iterative scrutiny of the data and discussion between TJ, KM, and RC. A summary of the common themes across the reviews, grouped under the three overarching themes, was then developed, including highlighting contrasting findings.

## Results

### Study selection

**Fig. 1:**
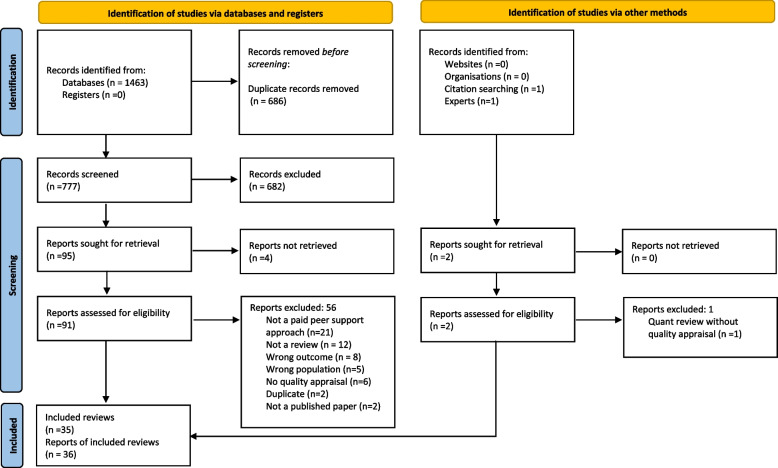
PRISMA flow diagram [29]

The search strategy identified 777 references to be screened (a further 2 papers were identified through other methods); 93 full text articles were assessed for eligibility with 57 excluded (see Additional file 1: Appendix 4 for reasons for exclusion). Thirty-five reviews (reported in 36 papers) were included (see Fig. 1).

### Characteristics of included reviews

Review characteristics are detailed in Table 1. Of the 35 included reviews, 13 were systematic reviews with meta-analyses, 13 were systematic reviews without meta-analyses, 3 were systematic reviews with a qualitative synthesis and 6 were scoping reviews. The individual reviews included between 95 and 40,927 participants; 6 reviews did not report the number of participants. For reviews where the population were service users, almost all were categorised as adults with mental health problems. Thirteen reviews specified that participants had severe mental illness (SMI) diagnoses [1, 21, 22, 35–45], six reviews explicitly included studies with participants accessing mental health services [22, 37, 38, 43, 45] [46], three reviews were conducted in perinatal populations [47–49], three reviews included participants with any/common mental health conditions [50–52], four reviews included participants with substance use disorders [1, 38, 53, 54], two reviews included participants with eating disorders [55, 56], one included people experiencing suicidality [57] and one included articles on peer support for crisis management [58]. The samples in the remaining reviews were PSWs and various stakeholders (e.g. non-peer staff, service users) [23, 24, 34, 59–64]. Most reviews included interventions involving any form of peer support, individual, group or combined, although three reviews looked at group peer support alone [35, 43, 49], and three reviews looked at individual peer support alone [1, 40, 45]. Reviews looked at peer support delivered in-person, online or over the phone, and surveyed a range of approaches including both structured and unstructured peer support (see Table 1). The reviews included 426 primary studies. We assessed study overlap; most primary studies (*n* = 300) were only included in one review; however, many primary studies were included twice (*n* = 72), three times (*n* = 18) to a maximum of nine times (*n* = 1) (see Additional file 1: Appendix 5 for overlapping studies). Only 1 review reported that people with lived experience were involved in the review [57]. Only 2 reviews assessed certainty of evidence (using GRADE) [21, 22].

**Table 1:** Characteristics of included studies

Reference	Review aim	*N* included studies (date range), geographical setting	Design of included primary studies	Population, *N*	Type of peer support	Format of peer support, setting	Quality appraisal tool, ratings	AMSTAR2
**Systematic review and meta-analyses**								
Burke et al. (2019) [51]	Effectiveness	*N* = 23 (2007–2017); USA (*n* = 16), Europe (*n* = 5), Canada (*n* = 1)	RCT (*n* = 15), pre-post (*n* = 8)	Adults with any mental health problem (and comprised ≥ 50% of sample) *N* = 6191	Individual, group, peer-run services	In-person Settings: Inpatient; Community/ outpatient MH	EPHPP Weak (10 studies), Moderate (9 studies), strong (4 studies)	Critically low
Chien et al. (2019) [21]	Effectiveness	*N* = 13 (2004–2017); USA (*n* = 8), UK (*n* = 1), Germany (*n* = 1), Netherlands (*n* = 2), China (*n* = 1)	RCT (all studies)	People (majority between 18 and 65 years) with schizophrenia or similar serious mental illness *N* = 2479	Individual, group	Structured (e.g. manualised interventions; psychoeducation) and unstructured (e.g. discussion on set topics) Settings: Inpatient; Community/outpatient MH	Cochrane RoB Tool > half had unclear RoB for the majority of domains; ~ a quarter had high RoB for 4 domains	High
Fang et al. (2022) [47]	Effectiveness	*N* = 16 (2000–2020); USA (*n* = 4), China (*n* = 4), Canada (*n* = 2), Pakistan (*n* = 2), India (*n* = 1), Zimbabwe (*n* = 1), Singapore (*n* = 1), Iran (*n* = 1)	RCT (all studies)	Pregnant women/women who gave birth within 1 year with diagnosis/risk of perinatal depression *N* = 3154	Group, individual, combination	In-person; phone; online; combination Settings: NR	Cochrane RoB Tool Overall RoB was low. One study had high RoB for random sequence generation, two studies had low RoB for blinding participants/personnel, three studies had high risk of other bias	Critically low
Fuhr et al. (2014) [37]	Effectiveness	*N* = 14 (1995–2012); USA (*n* = 9), Canada (*n* = 4), Netherlands (*n* = 1)	RCT (all studies)	Adult service users with an SMI or depression diagnosis *N* = 3595	Individual, group	In-person; telephone Structured (manual-based) Settings: Community/outpatient MH care	Cochrane RoB Tool 6 RCTs had overall high RoB	Low
Huang et al. (2020) [48]	Effectiveness	*N* = 10 (2000–2019); USA (*n* = 1), Canada (*n* = 3), China (*n* = 3), India (*n* = 1), Pakistan (*n* = 1), South Africa (*n* = 1)	RCT (all studies)	Pregnant women/women who gave birth within 1 year with diagnosis/risk of perinatal depression *N* = 3076	Group, individual, combination	In-person; telephone; combination (including internet) Settings: Inpatient and crisis (incl. Hospital); Community/outpatient MH care; Participant’s home or workplace	Cochrane RoB Tool 65% had low RoB, approximately 35% had unclear RoB	Critically low
Lloyd-Evans et al. (2014) [22]	Effectiveness	*N* = 18 (1982–2013); USA (*n* = 14), Canada (1), UK (*n* = 1), Netherlands (*n* = 1), Australia (*n* = 1)	RCT (all studies)	Adults with SMI diagnoses or those using secondary MH services *N* = 5597	Individual, group	In-person; online Structured (manualised), unstructured (e.g. befriending), combination Settings: Community/outpatient MH care	Cochrane RoB Tool All but 2 studies exhibited some RoB (unclear or high)	Low
Lyons et al. (2021) [35]	Effectiveness	*N* = 8 (2011–2018); USA (*n* = 7), Switzerland (*n* = 1)	RCT (all studies)	Adults with any mental health condition (including SMI) *N* = 2131	Group	Online; in-person Structured (manualised, classes), unstructured (*n* = 1; mutual support) Setting: NR	Cochrane RoB Tool Low RoB (*N* = 2 studies); high RoB (*N* = 2); unclear RoB (*N* = 4)	Critically low
Peck et al. (2023) [40]	Effectiveness	*N* = 17 (2009–2018); USA (*n* = 12), Canada (*n* = 1), Australia (*n* = 1), UK (*n* = 1), Netherlands (*n* = 1), Singapore (*n* = 1)	RCTs (*n* = 11), non-randomised controlled trial (*n* = 1), pre-test-post-test (*n* = 5)	Adults, majority diagnosed with schizophrenia, bipolar disorder or major affective disorders *N* = 3189	Individual	Peer-delivered self-management interventions or self-management education programmes incorporating elements of peer-assisted recovery Settings: NR	JBI critical appraisal for RCTs and Quasi-experimental trials RCTs: low-moderate quality; quasi-experimental trials: moderate quality	Critically low
Pitt et al. (2013, 2013) [41, 42]	Effectiveness	*N* = 11 (1979–2011); USA (*n* = 9), Australia (*n* = 1), UK (*n* = 1)	RCT (all studies)	Adults with severe mental health diagnoses *N* = 2796	Individual, group	Settings: community/outpatient MH care	Cochrane RoB Tool Study quality moderate to low. Most studies had: unclear RoB for random sequence generation, allocation concealment, high RoB blinded outcome assessment, selective reporting	Low
Smit et al. (2022) [52]	Effectiveness	*N* = 30 (2003–2020); USA (*n* = 18), UK (*n* = 2), Canada (*n* = 3), Netherlands (*n* = 3), Australia (*n* = 1), Singapore (*n* = 1), Switzerland (*n* = 1), Germany (*n* = 1)	RCT (all studies)	Adults with any mental health condition *N* = 4597	Individual, group	In-person; online Settings: NR	Cochrane RoB Tool High RoB (*N* = 21 studies), some concerns for RoB (*N* = 6), low RoB (*N* = 3)	Low
Sun et al. (2022) [43]	Effectiveness	*N* = 7 (*N* papers = 8) (2011–2021); USA (*n* = 5), Germany (*n* = 2), Switzerland (*n* = 1)	RCT (all studies)	People (age unspecified; final sample included adolescents and adults) with any mental health problem including mental health service users without reported diagnoses *N* = 763	Group	In-person Structured (e.g. Open, Honest, Proud classes) Settings: NR	Cochrane RoB Tool Studies were generally of low/moderate RoB	Critically low
Wang et al. (2022) [44]	Effectiveness	*N* = 28 (2004–2020); USA (*n* = 18), China (*n* = 5), Canada (*n* = 2), Netherlands (*n* = 2), Germany (*n* = 1)	RCT (all studies)	Adults: individuals or family members with serious mental illness *N* = 806 families *N* = 6572 individuals	Individual, family	Settings: inpatient and crisis (incl. Hospitals); community/outpatient MH care; hostels	Cochrane RoB Tool The majority of studies were at low risk of bias	Critically low
White et al. (2020) [45]	Effectiveness	*N* = 19 (*N* papers = 23) (1995–2018); USA (*n* = 12), UK (*n* = 3), Canada (*n* = 1), Australia (*n* = 1), Germany (*n* = 1), Japan (*n* = 1)	RCT (all studies)	Adults using mental health services with any diagnoses *N* = 3329	Individual	In-person; online; combination Structured (e.g. workbooks) and unstructured (e.g. mentoring) Settings: inpatient and crisis (incl. Hospital); community/outpatient MH care	Cochrane RoB Tool Overall quality of trials (compared to previous reviews) is low to moderate	Critically low
**Systematic review (without meta-analyses)**								
Bassuk et al. (2016) [53]	Effectiveness	*N* = 9 (2005–2013) USA	RCT (*n* = 4), quasi-experimental (*n* = 3), comparison group (*n* = 1), programme evaluation (no comparison) (*n* = 1)	People (age unspecified; final sample all adults) in recovery from addiction from alcohol and/or drugs *N* = 6883	NR	Settings: Inpatient; Community/outpatient MH services; third sector	EPHPP Methodologically strong (*n* = 2 studies), moderate (*n* = 2), weak (*n* = 5)	Critically low
Chinman et al. (2014) [1]	Effectiveness	*N* = 20 (N papers = 24) (1995–2013) International (countries not specified)	RCT (*n* = 11), quasi-experimental (*n* = 6), correlational or descriptive (*n* = 3)	Adults with SMI or co-occurring substance use disorders *N* = 40,927	Individual	Peers added to traditional services, peers assuming regular provider positions, peers delivering structured curricula Settings: Inpatient; Community/outpatient MH care	Criteria developed for the ‘assessing the evidence base series’, that this paper was a part of Limited (several methodological limitations) (*n* = 16), adequate (few or minor methodological limitations) (*n* = 4)	Critically low
du Plessis et al. (2020) [23]	Experiences	*N* = 24 (1998 – 2018); USA (*n* = 7), Canada (*n* = 2), Australia (*n* = 7), UK (*n* = 3), South Korea (*n* = 1), Hong Kong (*n* = 1), Unknown (*n* = 1) Multiple (*n* = 2)	Qualitative (*n* = 14), narrative (*n* = 6), mixed methods (*n* = 1), meta-synthesis (*n* = 1), literature review (*n* = 2)	PSWs (substance abuse or mental health) *N* = 307 (1 paper did not report sample size)	NR	Mental health (majority) and substance abuse settings	No quality appraisal	Critically low
Fortuna et al. (2020) [36]	Effectiveness	*N* = 30 (2005–2019); USA (*n* = 22), Australia (*n* = 5), Italy (*n* = 1), Japan (*n* = 1), Denmark (*n* = 1)	RCT (*n* = 11), quasi-experimental (*n* = 3), pre-post designs (*n* = 10), exploratory (*n* = 1), mixed methods (*n* = 1), qualitative (*n* = 2)	Adults with schizophrenia or bipolar disorder *N* = 4642	Individual, group	Digital peer support	MQRS High methodological quality (*n* = 6), low quality (*n* = 4)	Critically low
Gaiser et al. (2021) [38]	Effectiveness	*N* = 23 (2013–2020); USA	RCT (*n* = 1), quasi-experimental (*n* = 3), cohort analytic (*n* = 2), cross-sectional (*n* = 1), retrospective comparison group (*n* = 1), survey (*n* = 1)	Adults with mental health or substance use disorder or those with current or past use of MH or SUD services *N* = 14,098	Individual, group	Telephone; In-person Structured (following a manual/curriculum) and unstructured (without predetermined format – individualised participant needs) Settings: Community/ outpatient MH care; Inpatient and crisis (including hospitals); Participant’s home, third sector	EPHPP Weak (*n* = 12)	Critically low
Ibrahim et al. (2020) [24]	Implementation	*N* = 53 (1995–2018); USA (*n* = 30), UK (*n* = 7), Australia (*n* = 5), Canada (*n* = 3), Republic of Ireland (*n* = 2), Belgium (*n* = 1), Germany (*n* = 1), Hong Kong (*n* = 1), Japan (*n* = 1), Netherlands (*n* = 1), Israel & USA (*n* = 1)	RCTs (*n* = 10) Qualitative (*n* = 38), Cohort studies (*n* = 4) Control studies (*n* = 1)	PSWs supporting adults with mental illness *N* = NR	Individual, group, combination	Excluded online only Structured (e.g. health coaching, a ‘recovery’ training course), unstructured (e.g. PSWs sharing empathy, insights and skills) Settings: Community/outpatient MH care; Inpatient and crisis (incl. hospitals); third sector; other rehabilitation services	CASP Good quality (*n* = 47), Fair quality (*n* = 1), Poor quality (*n* = 5)	Critically low
Lewis & Foye (2022) [55]	Implementation, experiences	*N* = 10 (2006–2020); UK (*n* = 4), USA (*n* = 2), Australia (*n* = 2), Netherlands (*n* = 1), Australia, UK, USA, Canada (*n* = 1)	RCT (*n* = 6), quasi experimental (*n* = 4)	PSWs with lived experience of eating disorders *N* = 73 (N not reported in 4 studies) People with/at risk of eating disorders *N* = 4878	NR	In-person; online Various intervention content, e.g. sharing recovery narratives, providing guidance, deliver lessons in schools Settings: Inpatient and crisis (incl. Hospitals); community/outpatient MH care; participant’s home; schools, third sector	JBI Checklist for qualitative research 5 qualitative studies assessed: 8/10 (*n* = 3), 7/10 (*n* = 1), 6/10 (*n* = 1)	Critically low
Miyamoto & Sono (2012) [63]	Effectiveness, experiences	*N* = 51 (1988–2010) International (countries not specified)	Qualitative studies (*n* = 19), reviews (*n* = 8), other (*n* = NR)	PSWs supporting adults with mental health difficulties *N* = NR	NR	In-person, Setting: NR	No quality appraisal	Critically low
Mutschler et al. (2022) [46]	Implementation	*N* = 19 (2007–2019); USA (*n* = 12), Australia (*n* = 5), Scotland (*n* = 1), England (*n* = 1)	Mixed methods (*n* = 7), qualitative (*n* = 10), quantitative (*n* = 1), discussion paper (*n* = 1)	Individuals (age unspecified) seeking services for mental health. Diagnoses NR *N* = NR	Individual, group	Settings: inpatient and crisis (incl. Hospital); community/outpatient MH care; third sector; primary care	Studies rated on quality in terms of relevance to review, including attention to/use of implementation strategies Quality rating: high (*n* = 8), medium (*n* = 11), low (*n* = 0)	Critically low
Pellizzer & Wade (2022) [56]	Effectiveness	*N* = 11 (2014–2022) International (countries not specified)	RCTs (*n* = 4), Case study/series (*n* = 7)	People (age unspecified; final sample included adults and young people) with eating disorders (*N* = 1326) or carers (*N* = 289) Also included 2 families	Individual, combination	Peer-led or co-led/ adjunct treatment Structured programmes and unstructured (flexible content) Settings: NR	Cochrane RoB tool (RCTs) For all 4 RCTs: low risk rated for most items, all had items rated high risk or unclear risk Specified sub-selection of 2010 CONSORT guidelines Mean study quality: 6.45/9	Critically low
Reif et al. (2014) [54]	Effectiveness	*N* = 10 (1998–2011) International (countries not specified)	RCTs (*n* = 2), Quasi-experimental (*n* = 4), pre-post (*n* = 4)	Adults with substance use disorders *N* = 7203	Group, individual, combination	In-person, telephone Structured (e.g. coaching, counselling, activities, informational support, handouts) and unstructured (open-ended contact) Settings: various	Strength of the evidence (using criteria developed for the publication series) The evidence for peer support met the minimum criteria for the moderate category	Critically low
Triece et al. (2022) [50]	Effectiveness, implementation	*N* = 24 (2008–2021) LMICs: Uganda (*n* = 3), Ethiopia (*n* = 1), Zimbabwe (*n* = 1), Libya (*n* = 1), South Africa (*n* = 1), India (*n* = 2), Pakistan (*n* = 2), Philippines (*n* = 1), China (*n* = 1)	Qualitative (*n* = 7), pre-post case series (*n* = 3), RCTs (*n* = 7), mixed methods (*n* = 6), quasi-experimental (*n* = 1)	Adults with common mental disorders *N* = 4694	Individual, group, combined	Psychotherapeutic strategies e.g. psychoeducation, social/emotional support, problem-solving Settings: Community, clinic based	JBI Checklist for Qualitative Research; MMAT Case series were mostly low RoB. Mixed methods and qualitative design were overall low risk Cochrane RoB Tool: RCTs were mostly low RoB	Critically low
Vandewalle et al. (2016) [62]	Implementation	*N* = 18 (1998–2015); USA (*n* = 8), Canada (*n* = 3), England (*n* = 3), Australia (*n* = 2), New Zealand (*n* = 1), Netherlands (*n* = 1)	Qualitative (*n* = 15), mixed (*n* = 3)	PSWs (adults) employed in mental health services *N* = 470	NR	Settings: Inpatient and crisis (incl. hospitals); Community/outpatient MH care	CASP for qualitative studies Average of 25% of items were evaluated negatively in studies	Critically low
**Systematic review and qualitative synthesis**								
Bailie & Tickle (2015) [34]	Experiences	*N* = 8 (*N* papers = 10) (1996–2013); UK (*n* = 2), USA (*n* = 6), Australia (*n* = 1), Canada (*n* = 1)	Qualitative (all studies)	PSWs (for mental health) *N* = 96	NR	Settings: various including community/outpatient MH services	CASP for qualitative studies Variable study quality, scores ranged from lowest, 15 (*n* = 1 paper), to 34 (*n* = 1 paper)	Critically low
Jones et al. (2014) [49]	Experiences	*N* = 5 (1995–2012); England (*n* = 2), Finland (*n* = 1), Canada (*n* = 2)	Qualitative (all studies)	Women with perinatal mental illness *N* = 95	Group	NR	CASP for qualitative studies Overall, studies were of a reasonable quality	Critically low
Walker & Bryant (2013) [61]	Experiences	*N* = 25 (N papers = 27) (1994–2010); USA (*n* = 18), Canada (*n* = 4), Australia (*n* = 2), UK (*n* = 3)	Qualitative (*n* = 18), mixed methods (*n* = 5), case study (*n* = 2)	PSWs (*n* = 258), non-peer staff (*n* = 232), service users (*n* = 88) Total *N* = 578	NR	Settings: Statutory mental health settings, settings that share leadership with statutory mental health settings (taken from inclusion criteria)	CASP NR	Critically low
**Scoping review**								
Akerblom & Ness (2023) [60]	Effectiveness	*N* = 172 (2010–2021); USA (*n* = 75), Oceania (*n* = 36), GB (*n* = 24), Canada (*n* = 15), Europe excluding GB (*n* = 19), Asia (*n* = 8), Southern America (*n* = 1)	NR	Studies of mental health and substance use PSW roles (variety of stakeholders, e.g. PSWs, carers, non-peer staff) *N* = 12,044 (*N* not reported in 45 studies)	NR	Settings: adult mental health and substance use services	No quality appraisal	Critically low
Bowersox et al. (2021) [58]	Effectiveness	*N* = 84 (1968–2019); International (countries not specified)	NR	Articles on mental health peer support for suicide prevention/crisis management with adults *N* = NR	NR	Telephone; online; in-person Setting: inpatient and crisis (incl. hospitals), community/outpatient MH services, third sector	Quality evaluated based on United States Preventative Services Task Force guidelines 3.6% highest quality, 31% lowest level	Critically low
Ong et al. (2022) [39]	Implementation	*N* = 16 (2012–2021); Asia, majority in India (*n* = 5) and Hong Kong (*n* = 4)	RCT (*n* = 1), mixed methods (*n* = 3), commentary/editorials (*n* = 4), thesis (*n* = 1), ethnographic (*n* = 1), qualitative (*n* = 5), cross-sectional (*n* = 1)	People (any age; final sample included adults and children) with any mental health condition (including SMI) *N* = 528 participants *N* = 82 service providers	Individual, group	In-person; telephone Individual: unstructured conversations, development of recovery plans, outreach programmes Group: discussions, structured activities, e.g. role-plays, exercise, homework Settings: Community/outpatient MH care; inpatient and crisis (incl. Hospitals)	No quality appraisal	Critically low
Schlichthorst et al. (2020) [57]	Effectiveness	*N* = 7 (*N* papers = 8) (2006–2019); USA (*n* = 3), Germany (*n* = 1), Germany/Austria (*n* = 1), China (*n* = 1), Australia (*n* = 1)	Cross-sectional (*n* = 1), qualitative (*n* = 1), descriptive (*n* = 3), RCT (*n* = 1), survey (*n* = 1)	People (age unspecified) who experience suicidality *N* = NR	Individual, group	In-person; online, structured (e.g. PSWs provide training in community) and unstructured (e.g. 1–1 peer support with flexible frequency/duration) Settings: inpatient and crisis (incl. Hospitals); community/outpatient MH care; schools; online	No quality appraisal	Critically low
Viking et al. (2022) [64]	Experiences	*N* = 22 (*N* papers = 23) (2011–2021); UK (*n* = 8), Canada (*n* = 2), Switzerland (*n* = 1), Australia (*n* = 3), Belgium (*n* = 1), USA (*n* = 3), German (*n* = 3), Canada & Norway (*n* = 1), Norway & USA (*n* = 1)	Qualitative (*n* = 21), quantitative (*n* = 1)	Literature concerning PSWs in mental healthcare PSW (*n* = 235), service users (*n* = 18), non-peer staff (*n* = 191), mixed (*n* = 247)	NR	Settings: Formal MH care settings	No quality appraisal	Critically low
Zeng & McNamara (2021) [59]	Implementation	*N* = 28 (2006–2020); USA (*n* = 12), UK (*n* = 7), Australia (*n* = 8), Canada (*n* = 1)	Qualitative (*n* = 25), mixed methods (*n* = 3)	Mental health PSWs *N* = NR	NR	Settings: statutory MH services; third sector	No quality appraisal	Critically low

*CASP* Critical Appraisal Skills Programme, *Combination* group + individual peer support, *EPHPP* The Effective Public Health Practice Project tool, *JBI* Joanna Briggs Institute, *LMICs* low- and middle-income countries, *MH* mental health, *Mixed sample* PSWs, non-peer staff, service users, commissioners, policy makers, *MMAT* Mixed Methods Appraisal Tool, *MQRS* Methodological Quality Rating Scale, *NR* not reported, *RCT* randomised controlled trials, *RoB* Risk of Bias, *PSW* peer support worker, *SMI* severe mental illness

### Quality appraisal of included reviews

Most reviews were appraised as low or critically low (97%) quality and one review was appraised as high quality. The most common weaknesses were in critical domains concerning registering protocols before commencement of the review (21 studies), justification of excluding individual studies (28 studies) and considering risk of bias when interpreting results (13 studies). Reviews without meta-analyses were not scored in the critical domains assessing meta-analytical method or publication bias. There were 13 studies with meta-analyses assessed in these two domains: two of these exhibited one critical weakness and two exhibited two critical weaknesses. As scoping reviews are intended to provide overviews of existing literature regardless of risk of bias [65], scoping reviews were not scored in the critical domain concerning risk of bias assessment techniques (see Additional file 1: Appendix 3 for adjustments to quality appraisal for scoping and qualitative reviews). Of the 29 reviews that were eligible to be scored in this domain, 10 exhibited a critical weakness. The review eliciting high confidence was a Cochrane review [21]. No reviews were rated as moderate. AMSTAR 2 ratings are detailed in Table 1 and in full in Additional file 1: Appendix 3.

### Results of synthesis

### RQ1: What is the effectiveness (e.g. clinical, social, functional) and cost-effectiveness of paid peer support approaches for mental health?

Effectiveness outcomes were reported in 23 reviews (66% of total). A wide variety of clinical, recovery and psychosocial effectiveness outcomes were reported across both meta-analysis [21, 22, 37, 40–45, 47, 48, 51, 52] and narrative results [1, 21, 22, 35–38, 40–44, 48, 50, 51, 53, 54, 56–58, 60]. Comparator groups also varied across the primary studies included in the reviews, including Treatment as Usual (TaU), active controls (e.g. a comparable standard treatment) and waitlist control groups.

All outcomes except for one (family or carer use of formal community support services; [44]) were service user outcomes, rather than carer, staff or PSW outcomes. Outcomes from systematic reviews with meta-analysis are reported in Tables 2, 3 and 4. Effectiveness results from reviews not including meta-analysis are summarised at the end of this section and reported in full in Additional file 1: Appendix 6. Evidence was heterogenous across all outcomes and reviews, with many analyses reporting no effect. In the meta-analysis results, there was often notable heterogeneity. There was limited data on cost and cost-effectiveness, but the evidence available from three systematic reviews without meta-analyses (See Additional file 1: Appendix 6) suggested that peer support interventions were low cost and cost-saving [38, 48, 50].

**Table 2:** Meta-analyses effectiveness results: clinical outcomes

Author (year)	Outcome	*N* of studies (*N* of participants)	Population	Effect measure	Effect size (95% CI), *p*-value	Heterogeneity, *I*^2^, 95% CI, *χ*^2^, df	AMSTAR2	Summary findings
Depression								
Fang et al. (2022) [47]	Perinatal depression (various end time points)	16 (3154)	Pregnant women/ women who gave birth within 1 year with diagnosis/ risk of perinatal depression	SMD	− 0.39 (− 0.54, − 0.24); *Z* = 9.42, *p* < 0.00001 (results taken from text)	*I*^2^ = 78%; *χ*^2^ = 91.38 (df = 20, *p* < 0.00001)	Critically low	**Significant reduction in perinatal depression**
Huang et al. (2020) [48]	Depression (post-intervention)	9 (1617)	Pregnant women/ women who gave birth within 1 year with diagnosis /risk of perinatal depression	SMD, Z	− 0.37 (− 0.66, − 0.08), *p* = 0.01 *Z* = 2.47 (*p* = 0.01)	*I*^2^ = 84%, *p* < 0.00001, Tau^2^ = 0.14; *χ*^2^ = 49.37 (df = 8, *p* < 0.00001)	Critically low	**Significant reduction in perinatal depression scores**
Huang et al. (2020) [48]	Depression ‘events’ (binary measure—post-intervention)	7 (1644)	Pregnant women/ women who gave birth within 1 year with diagnosis /risk of perinatal depression	RR, Z	0.69 (0.49, 0.96), *p* = 0.03 *Z* = 2.22 (*p* = 0.03)	*I*^2^ = 70%, *p* = 0.003 Tau^2^ = 0.11; *χ*^2^ = 20.25 (df = 6; *p* = 0.003)	Critically low	**Significant reduction in risk of perinatal depression**
Lloyd-Evans et al. (2014) [22]	Depression and anxiety (post-intervention)	3 (861)	Adults with SMI or those using secondary MH services	SMD	− 0.10 (− 0.24, 0.03)	*I*^2^ = 0%, *χ*^2^ = 1.97 (*p* = 0.37)	Low	No effect
Lyons et al. (2021) [35] ^b^	Depression (post-intervention)	4 (929)	Adults with any mental health condition (including SMI)	SMD	− 0.09 (− 0.22, 0.04), *p* = 0.18	*I*^2^ = 0%, *χ*^2^ = 1.11 (df = 2)	Critically low	No effect
Sun et al. (2022) [43] ^b^	Depression (end of treatment)	5 (372)	Adults and adolescents with any mental health problem including MH service users without reported diagnoses	SMD	− 0.05 (− 0.26, 0.15), *p* = 0.34	*I*^2^ = 0%, *χ*^2^ = 1.62 (df = 1, *p* = 0.76)	Critically low	No effect
Depression: follow-up								
Lloyd-Evans et al. (2014) [22]	Depression and anxiety (6 months follow-up)	2 (721)	Adults with SMI or those using secondary MH services	SMD	− 0.17 (− 0.32, − 0.03)	*I*^2^ = 0%, *χ*^2^ = 0.15 (*p* = 0.70)	Low	**Significant reduction in depression and anxiety**
Lyons et al. (2021) [35] ^b^	Depression (3–6 months follow-up)	3 (674)	Adults with any mental health condition (including SMI)	SMD	− 0.12 (− 0.27, 0.03), *p* = 0.11	*I*^2^ = 0%, *χ*^2^ = 0.95 (df = 2)	Critically low	No effect
Clinical recovery								
Smit et al. (2022) [52]	Clinical recovery (post-intervention)	22 (NR)	Adults with any mental health diagnosis	Hedges’ g	0.19 (0.11–0.27), *p* < 0.001	*I*^2^ = 10% (95% CI 0–44)	Low	**Significant improvement in clinical recovery**
Clinical recovery: follow-up								
Smit et al. (2022) [52]	Clinical recovery (6–9 months follow-up)	13 (NR)	Adults with any mental health diagnosis	Hedges’ g	0.17 (0.08–0.26), *p* = 0.002	*I*^2^ = 0% (95% CI 0–57)	Low	**Significant improvement in clinical recovery**
Smit et al. (2022) [52]	Clinical recovery (12–18 months follow-up)	8 (NR)	Adults with any mental health diagnosis	Hedges’ g	0.10 (− 0.21,0.40), *p* = 0.48	*I*^2^ = 63% (95% CI 20–83)	Low	No effect
Mental health symptoms								
Peck et al. (2023) [40] ^a^	Symptom severity (time point not stated)	5 (1094)	Adults, majority diagnosed with schizophrenia, bipolar disorder or major affective disorders	SMD, Z	− 0.30 (− 0.55, − 0.04), *Z* = 2.29 (*p* = 0.02)	*I*^2^ = 75%, Tau^2^ = 0.06, *χ*^2^ = 15.77 (df = 4, *p* = .003)	Critically low	**Significant reduction in symptom severity**
Wang et al. (2022) [44]	Psychotic symptoms (post-family-led peer support)	3 (742)	Adult individuals or family members with SMI	SMD, Z	− 1.45 (− 2.68, − 0.22), *Z* = 2.32, *p* = 0.02	*I*^2^ = 98%, Tau^2^ = 1.92; *χ*^2^ = 189.37 (df = 4, *p* < 0.00001)	Critically low	**Significant reduction in psychotic symptoms**
Lloyd-Evans et al. (2014) [22]	Overall psychiatric symptoms (post-treatment)	3 (753)	Adults with SMI or those using secondary MH services	SMD	− 0.07 (− 0.39, 0.24)	*I*^2^ = 74%, *χ*^2^ = 7.83 (*p* = 0.02)	Low	No effect
Lloyd-Evans et al. (2014) [22]	Symptoms of psychosis (post-treatment)	2 (696)	Adults with SMI or those using secondary MH services	SMD	− 0.08 (− 0.27, 0.03)	Not reported	Low	No effect
Lyons et al. (2021) [35] ^b^	Global symptoms (post-intervention)	3 (823)	Adults with any mental health condition (including SMI)	SMD	− 0.13 (− 0.27, 0.01), *p* = 0.07	*I*^2^ = 0%, *χ*^2^ = 1.11 (df = 2)	Low	No effect
Pitt et al. (2013) [41, 42]	Mental health symptoms (time point not stated)	2 (197)	Adults with severe mental health diagnoses	SMD, Z	− 0.24 (− 0.52, 0.05), *Z* = 1.65 (*p* = 0.1)	*I*^2^ = 0%, Tau^2^ = 0; *χ*^2^ = 0.52 (df = 2; *p* = 0.77)	Low	No effect
Sun et al. (2022) [43] ^b^	Anxiety (post-intervention)	2 (175)	Adults and adolescents with any mental health problem including MH service users without reported diagnoses	SMD	0.29 (− 0.01, 0.58), *p* = 0.06	*I*^2^ = 0%, *χ*^2^ = 0.09 (df = 1, *p* = 0.76)	Critically low	No effect
Wang et al. (2022) [44]	Psychotic symptoms (post individual-led peer support)	11 (2651)	Adult individuals or family members with SMI	SMD	–0.30 (–0.73, 0.13), *Z* = 1.38, *p* = 0.17	*I*^2^ = 96%; Tau^2^ = 0.5; *χ*^2^ = 270.29 (df = 10, *p* < .00001)	Critically low	No effect
Mental health symptoms: follow-up								
White et al. (2020) [45]^a^	Psychiatric symptoms (6–24 months follow-up)	6 (857)	Adults using mental health services with any diagnoses	SMD, Z	− 0.01 (− 0.21, 0.20), *Z* = 0.0 (*p* = 0.961)	*I*^2^ = 53%, *χ*^2^ = 10.7, *p* = 0.057	Critically low	No effect
Service use								
Wang et al. (2022) [44]	Rehospitalisation (last follow-up)	3 (483)	Adult individuals or family members with SMI	SMD, Z	− 1.34 (− 1.94, − 0.75), *Z* = 4.44, *p* < 0.00001	*I*^2^ = 87%, Tau^2^ = 0.32; *χ*^2^ = 23.15 (df = 3, *p* < 0.0001)	Critically low	**Significant reduction in rehospitalisation**
Wang et al. (2022) [44]	Duration of hospitalisation (last follow-up)	3 (483)	Adult individuals or family members with SMI	SMD, Z	− 1.48 (− 2.56, − 0.41), *Z* = 2.70, *p* = 0.007	*I*^2^ = 96%, Tau^2^ = 1.14; *χ*^2^ = 70.97 (df = 3, *p* < 0.0001)	Critically low	**Significant reduction in hospital duration**
Wang et al. (2022) [44]	Family/carer use of formal community support services (last follow-up)	4 (483)	Adult individuals or family members with SMI	SMD, Z	− 1.38 (2.19, − 0.56), *Z* = 3.32, *p* = 0.0009	*I*^2^ = 93%, Tau^2^ = 0.64, *χ*^2^ = 42.21 (df = 3, *p* < 0.00001)	Critically low	**Significant reduction in use of community support services**
Lloyd-Evans et al. (2014) [22]	Duration of admission (post-treatment)	3 (255)	Adults with SMI or those using secondary MH services	SMD	− 0.22 (− 0.72, 0.28) *p* = 0.03	*I*^2^ = 72%, *χ*^2^ = 7.16	Low	No effect
Pitt et al. (2013) [41, 42]	Length of stay (time point not stated)	2 (119)	Adults with severe mental health diagnoses	MD, Z	− 13.41 (− 32.09, 5.27), *Z* = 1.41 (*p* = 0.16)	*I*^2^ = 28.6%, Tau^2^ = 89.38; *χ*^2^ = 1.4 (df = 1, *p* = 0.24)	Low	No effect
Service use: follow-up								
White et al. (2020) [45]^a^	Risk of hospitalisation (3–24 months follow-up)	5 (497)	Adults using mental health services with any diagnoses	RR, Z	0.86 (0.66, 1.13), *Z* = 1.1 (*p* = 0.27)	*I*^2^ = 38%, *χ*^2^ = 6.5, *p* = 0.170	Critically low	**Significant reduction in risk of hospitalisation**
White et al. (2020) [45]^a^	Days in hospital (9–24 months follow-up)	5 (453)	Adults using mental health services with any diagnoses	SMD, Z	− 0.10 (− 0.34, 0.14), *Z* = 0.8, *p* = 0.426	*I*^2^ = 39%, *χ*^2^ (Q) = 10.7, *p* = 0.057	Critically low	No effect
Other clinical outcomes								
Sun et al. (2022) [43]^b^	Help-seeking (post-intervention)	2 (114)	Adults and adolescents with any mental health problem including MH service users without reported diagnoses	SMD	0.46 (0.10, 0.82), *p* = 0.01	*I*^2^ = 0%, *χ*^2^ = 0.93 (df = 1, *p* = 0.34)	Critically low	**Significant improvement in help-seeking**
Sun et al. (2022) [43]^b^	Disclosure-related distress (post-intervention)	2 (170)	Adults and adolescents with any mental health problem including MH service users without reported diagnoses	SMD	− 0.53 (− 0.84, − 0.23), *p* = 0.0006	*I*^2^ = 0%, *χ*^2^ = 0.25 (df = 2, *p* = 0.61)	Critically low	**Significant decrease in disclosure-related distress**
Wang et al. (2022) [44]	Medication adherence (last follow-up)	5 (371)	Adult individuals or family members with SMI	SMD	− 0.22 (− 0.43, − 0.01); *Z* = 2.08, *p* = 0.04	*I*^2^ = 0%; Tau^2^ = 0; *χ*^2^ = 1.54 (df = 4, *p* = 0.82)	Critically low	**Significant improvement in medical adherence**
Wang et al. (2022) [44]	Activation (last follow-up)	3 (375)	Adult individuals or family members with SMI	SMD	0.43 (0.19, 0.67); *Z* = 3.46, *p* = 0.0005	*I*^2^ = 19%; Tau^2^ = 0.01; *χ*^2^ = 2.47 (df = 2, *p* = 0.29)	Critically low	**Significant improvement in activation**
Sun et al. (2022) [43]^b^	Disclosure-related withdrawal (post-intervention)	3 (281)	Adults and adolescents with any mental health problem including MH service users without reported diagnoses	SMD	− 0.10 (− 0.33, 0.14), *p* = 0.42	*I*^2^ = 37%, *χ*^2^ = 3.20 (df = 2, *p* = 0.20)	Critically low	No effect
Wang et al. (2022) [44]	Alcohol use (last follow-up)	3 (257)	Adult individuals or family members with SMI	SMD	− 0.23 (− 0.49, 0.03); *Z* = 1.73, *p* = 0.08	*I*^2^ = 0%; Tau^2^ = 0; *χ*^2^ = 1.36 (df = 2, *p* = 0.51)	Critically low	No effect
Other clinical outcomes: follow-up								
Chien et al. (2019) [21]	Activation (medium term, 1–6 months follow-up)	3 (295)	Adults with schizophrenia or similar SMI	MD, Z	3.68 (− 1.85, 9.22), *Z* = 1.3 (*p* = 0.19)	*I*^2^ = 80.32, Tau^2^ = 18.09, *χ*^2^ = 10.16 (df = 2, *p* = 0.01)	High	No effect

^a^Review included studies of individual peer support only

^b^Review included studies of group peer support only; no stars = Review included studies of either individual or group peer support, or both

**Table 3:** Meta-analyses effectiveness results: recovery outcomes

**Author (year)**	**Outcome**	***N* of studies (*N* of participants)**	**Population**	**Effect measure**	**Effect size (95% CI), *p*-value**	**Heterogeneity, *I***^**2**^**, 95% CI,** *χ*^**2**^**, df**	**AMSTAR2**	**Summary findings**
Recovery								
Lloyd-Evans et al. (2014) [22]	Self-rated recovery (post-intervention)	4 (1066)	Adults with SMI or those using secondary MH services	SMD	− 0.24 (− 0.39, − 0.09)	*I*^2^ = 27%, *χ*^2^ = 4.09 (*p* = 0.25)	Low	**Significant improvement in self-rated recovery**
Lyons et al. (2021) [35]^b^	Recovery (post-intervention)	5 (1265)	Adults with any mental health condition (including SMI)	SMD	0.18 (0.07, 0.29), *p* = 0.002	*I*^2^ = 0%, *χ*^2^ = 4.01 (df = 4)	Critically low	**Significant improvement in recovery post-intervention**
Peck et al. (2023) [40]^a^	Self-perceived recovery (time frame not stated)	6 (1254)	Adults, majority diagnosed with schizophrenia, bipolar disorder or major affective disorders	SMD, Z	0.29 (0.12, 0.46), *Z* = 3.33 (*p* = 0.0009)	*I*^2^ = 48%, Tau^2^ = 0.02; *χ*^2^ = 9.65 (df = 5, *p* = 0.09);	Critically low	**Significant improvement in participants’ self-perceived recovery**
Wang et al. (2022) [44]	Recovery (last follow-up)	6 (1385)	Adult individuals or family members with SMI	SMD	0.21 (0.05, 0.36); *Z* = 2.67, *p* = 0.008	*I*^2^ = 41%; Tau^2^ = 0.01; *χ*^2^ = 8.46 (df = 5, *p* = 0.13)	Critically low	**Significant improvement in recovery**
Sun et al. (2022) [43]^b^	Recovery (post-intervention)	3 (197)	Adults and adolescents with any mental health problem including MH service users without reported diagnoses	SMD	0.14 (− 0.14, 0.42), *p* = 0.34)	*I*^2^ = 0%, *χ*^2^ = 0.43 (df = 2, *p* = 0.81)	Critically low	No effect
Recovery: follow-up								
Lloyd-Evans et al. (2014) [22]	Self-rated recovery (6 months follow-up)	2 (757)	Adults with SMI or those using secondary MH services	SMD	− 0.23 (− 0.37, − 0.09)	*I*^2^ = 0%, *χ*^2^ = 0.77 (*p* = 0.40)	Low	**Significant improvement in self-rated recovery**
Lyons et al. (2021) [35]^b^	Recovery (3–6 months follow-up)	4 (983)	Adults with any mental health condition (including SMI)	SMD	0.21 (0.08, 0.34), *p* = 0.002	*I*^2^ = 5%, *χ*^2^ = 3.16 (df = 3)	Critically low	**Significant improvement in recovery**
White et al. (2020) [45]^a^	Recovery (12–18 months follow-up)	3 (593)	Adults using mental health services with any diagnoses	SMD, Z	0.22 (0.01, 0.42), *Z* = 2.04, *p* = 0.04	*I*^2^ = 38%, Tau^2^ = 0.01; *χ*^2^ = 3.11 (df = 2, *p* = 0.21)	Critically low	**Significant improvement in recovery**
Chien et al. (2019) [21]	Recovery (medium term, 1–6 months follow-up)	3 (557)	Adults with schizophrenia or similar SMI	MD, Z	2.69 (− 0.82,6.20), *Z* = 1.5 (*p* = 0.13)	*I*^2^ = 33.33%, Tau^2^ = 3.52, *χ*^2^ = 3 (df = 2, *p* = 0.22)	High	No effect
Personal recovery								
Smit et al. (2022) [52]	Personal recovery (post-intervention)	19 (NR)	Adults with any mental health diagnosis	Hedges’ g	0.15 (0.04–0.27), *p* = 0.01	*I*^2^ = 43% (95% CI 1–67)	Low	**Significant improvement in personal recovery**
Personal recovery: follow-up								
Smit et al. (2022) [52]	Personal recovery (6–9 months follow-up)	12 (NR)	Adults with any mental health diagnosis	Hedges’ g	0.10 (− 0.10, 0.30), *p* = 0.28	*I*^2^ = 64% (95% CI 32–81)	Low	No effect
Smit et al. (2022) [52]	Personal recovery (12–18 months follow-up)	7 (NR)	Adults with any mental health diagnosis	Hedges’ g	0.54 (− 0.33, 1.41), *p* = 0.18	*I*^2^ = 93 (95% CI 89–96)	Low	No effect
Functional recovery								
Smit et al. (2022) [52]	Functional recovery (post-intervention)	25 (NR)	Adults with any mental health diagnosis	Hedges’ g	0.08 (− 0.02, 0.18), *p* = 0.11	*I*^2^ = 36% (95% CI 0–61)	Low	No effect
Functional recovery: follow-up								
Smit et al. (2022) [52]	Functional recovery (6–9 months follow-up)	17 (NR)	Adults with any mental health diagnosis	Hedges’ g	0.14 (0.01, 0.27), *p* = 0.03	*I*^2^ = 39% (95% CI 0–66)	Low	**Significant improvement in functional recovery**
Smit et al. (2022) [52]	Functional recovery (12–18 months follow-up)	10 (NR)	Adults with any mental health diagnosis	Hedges’ g	0.38 (− 0.21, 0.98), *p* = 0.18	*I*^2^ = 91% (95% CI 85–94)	Low	No effect

^a^Review included studies of individual peer support only

^b^Review included studies of group peer support only; no stars = Review included studies of either individual or group peer support, or both

**Table 4:** Meta-analyses effectiveness results: psychosocial outcomes

**Author (year)**	**Outcome**	***N* of studies (*N* of participants)**	**Population**	**Effect measure**	**Effect size (95% CI), *p*-value**	**Heterogeneity, *I***^**2**^**, 95% CI,** *χ*^**2**^**, df**	**AMSTAR2**	**Summary findings**
Hope								
Fuhr et al. (2014) [37]	Hope (up to 6 months follow-up)	3 (967)	Adult service users with an SMI or depression diagnosis	SMD, Z	0.24 (0.02, 0.46), *Z* = 2.16 (*p* = 0.03)	*I*^2^ = 65%, Tau^2^ = 0.02; *χ*^2^ = 5.74 (df = 2, *p* = 0.06)	Low	**Significant improvement in hope**
Lloyd-Evans et al. (2014) [22]	Hope (post-intervention)	4 (1072)	Adults with SMI or those using secondary MH services	SMD	− 0.14 (− 0.27, − 0.02)	*I*^2^ = 7%, *χ*^2^ = 3.21 (*p* = 0.36)	Low	**Significant improvement in hope**
Peck et al. (2023) [40]^a^	Hopefulness (time frame not stated)	6 (1155)	Adults, majority diagnosed with schizophrenia, bipolar disorder or major affective disorders	SMD, Z	0.31 (0.13, 0.49), *Z* = 3.35 (*p* = 0.0008)	*I*^2^ = 46%, Tau^2^ = 0.02, *χ*^2^ = 9.30 (df = 5, *p* = 0.10)	Critically low	**Significant improvement in hopefulness**
Wang et al. (2022) [44]	Hope (last follow-up)	4 (1000)	Adult individuals or family members with SMI	SMD	0.27 (0.06, 0.49); *Z* = 2.51, *p* = 0.01	*I*^2^ = 58%; Tau^2^ = 0.03; *χ*^2^ = 7.16 (df = 3; *p* = 0.07)	Critically low	**Significant improvement in hope**
Lyons et al. (2021) [35]^b^	Hope (post-intervention)	3 (1029)	Adults with any mental health condition (including SMI)	MD	0.18 (− 0.34, 0.69), *p* = 0.50	*I*^2^ = 0%, *χ*^2^ = 1.68 (df = 2)	Critically low	No effect
Sun et al. (2022) [43]^b^	Hopelessness (post-intervention)	2 (114)	Adults and adolescents with any mental health problem including MH service users without reported diagnoses	SMD	− 0.16 (− 0.52, 0.19), *p* = 0.37	*I*^2^ = 0%, *χ*^2^ = 0 (df = 1, *p* = 0.99)	Critically low	No effect
Hope: follow-up								
Lloyd-Evans et al. (2014) [22]	Hope (3–6 month follow-up)	3 (967)	Adults with SMI or those using secondary MH services	SMD	− 0.24 (− 0.46, − 0.02)	*I*^2^ = 65%, *χ*^2^ = 5.74 (*p* = 0.06)	Low	**Significant improvement in hope**
Chien et al. (2019) [21]	Hope SHS scale, medium term (1–6 months)	2 (789)	Adults with schizophrenia or similar SMI	MD, Z	0.37 (− 0.22,0.96), *Z* = 1.22, *p* < 0.22	*I*^2^ = 0%, Tau^2^ = 0; *χ*^2^ = 0.01 (df = 1, *p* = 0.91)	High	No effect
Chien et al. (2019) [21]	Hope SHS scale, long term (> 6 months)	3 (809)	Adults with schizophrenia or similar SMI	MD, Z	0.41 (− 0.15, 0.97), *Z* = 1.44 (*p* = 0.15)	*I*^2^ = 0%, Tau^2^ = 0, *χ*^2^ = 1.48 (df = 2, *p* = 0.48)	High	No effect
Empowerment								
Burke et al. (2019) [51]	Empowerment (group intervention, end of treatment)	5 (923)	Adults (including veterans) with any mental health problem	Hedges’ g	0.19 (0.03, 0.36), *p* = 0.02	*I*^2^ = 30%, Tau^2^ = 0.01, *Q* = 5.69, *p* = 0.22	Critically low	**Significant improvement in empowerment end of treatment**
Peck et al. (2023) [40]^a^	Empowerment (time frame not stated)	3 (348)	Adults, majority diagnosed with schizophrenia, bipolar disorder or major affective disorders	SMD, Z	0.46 (0.25, 0.67), *Z* = 4.20 (*p* < .0001)	*I*^2^ = 0%, Tau^2^ = 0; *χ*^2^ = 1.69 (df = 2, *p* = 0.43)	Critically low	**Significant improvement in empowerment**
Lloyd-Evans et al. (2014) [22]	Empowerment (post-intervention)	2 (286)	Adults with SMI or those using secondary MH services	SMD	− 2.67 (− 7.35, 2.02)	*I*^2^ = 97%, *χ*^2^ = 38.87 (p < 0.001)	Low	No effect
Lyons et al. (2021) [35]^b^	Empowerment (post-intervention)	4 (750)	Adults with any mental health condition (including SMI)	SMD	0.17 (− 0.07, 0.40), *p* = 0.17	*I*^2^ = 55%, *χ*^2^ = 6.67 (df = 3)	Critically low	No effect
Sun et al. (2022) [43]^b^	Empowerment (post-intervention)	3 (245)	Adults and adolescents with any mental health problem including MH service users without reported diagnoses	SMD	0.24 (− 0.01, − 0.50), *p* = 0.06	*I*^2^ = 0%, *χ*^2^ = 0.86 (df = 2, *p* = 0.65)	Critically low	No effect
Wang et al. (2022) [44]	Empowerment (last follow-up)	4 (2380)	Adult individuals or family members with SMI	SMD	–0.37 (–0.91, 0.17); *Z* = 1.34, *p* = 0.18	*I*^2^ = 94%; Tau^2^ = 0.24; *χ*^2^ = 52.72 (df = 3, *p* < .00001)	Critically low	No effect
Empowerment: follow-up								
Lloyd-Evans et al. (2014) [22]	Empowerment (6 months follow-up)	2 (538)	Adults with SMI or those using secondary MH services	SMD	− 0.25 (− 0.43, − 0.07)	*I*^2^ = 12%, *χ*^2^ = 1.13 (*p* = 0.29)	Low	**Significant improvement in empowerment**
White et al. (2020) [45]^a^	Empowerment (6–12 months follow-up)	4 (519)	Adults using mental health services with any diagnoses	SMD, Z	0.23 (0.04, 0.42), *Z* = 2.31, *p* = 0.02	*I*^2^ = 14%, Tau^2^ = 0.01; *χ*^2^ = 3.48 (df = 3, *p* = 0.32)	Critically low	**Significant improvement in empowerment**
Lyons et al. (2021) [35]^b^	Empowerment (3 weeks–6 months follow-up)	4 (750)	Adults with mental health conditions	SMD	0.17 (− 0.07, 0.40), *p* = 0.17	*I*^2^ = 55%, *χ*^2^ = 6.67 (df = 3)	Critically low	No effect
Quality of life								
Fuhr et al. (2014) [37]	Quality of Life (up to 6 months follow-up)	2 (639)	Adult service users with an SMI or depression diagnosis	SMD, Z	0.24 (0.08, 0.40), *Z* = 3.02 (*p* = .003)	*I*^2^ = 0%, Tau^2^ = 0, *χ*^2^ = 0 (df = 1, *p* = .98)	Low	**Significant improvement in quality of life**
Wang et al. (2022) [44]	Quality of life (last follow-up)	11 (2397)	Adult individuals or family members with SMI	SMD	0.14 (0.06, 0.22); *Z* = 3.45, *p* = 0.0006)	*I*^2^ = 0%; Tau^2^ = 0; *χ*^2^ = 4.59 (df = 10, *p* = 0.92)	Critically low	**Significant improvement in quality of life**
Lloyd-Evans et al. (2014) [22]	Quality of life (post-intervention)	5 (1039)	Adults with SMI or those using secondary MH services	SMD	0.04 (− 0.16, 0.24)	*I*^2^ = 52%; *χ*^2^ = 8.38 (*p* = 0.08)	Low	No effect
Quality of life: follow-up								
Lloyd-Evans et al. (2014) [22]	Quality of life (3–6 months follow-up)	2 (639)	Adults with SMI or those using secondary MH services	SMD	− 0.24 (− 0.40, − 0.08)	*I*^2^ = 0%, *χ*^2^ = 0.00 (*p* = 0.98)	Low	No effect
White et al. (2020) [45]^a^	Quality of life (12–24 months follow-up)	5 (688)	Adults using mental health services with any diagnoses	SMD, Z	0.08 (− 0.11, 0.26), *Z* = 0.8 (*p* = 0.424)	*I*^2^ = 32%, *χ*^2^ = 5.9 (*p* = 0.206)	Critically low	No effect
Satisfaction with care								
Lloyd-Evans et al. (2014) [22]	Satisfaction (post-intervention)	3 (332)	Adults with SMI or those using secondary MH services	SMD	0.02 (− 0.02, 0.23)	*I*^2^ = 0%, *χ*^2^ = 0.95 (*p* = 0.62)	Low	No effect
Pitt et al. (2013) [41, 42]	Satisfaction with treatment (PSW in a professional role, time point not stated)	2 (213)	Adults with severe mental health diagnoses	SMD, Z	− 0.22 (− 0.69, 0.25), *Z* = 0.93 (*p* = 0.35)	*I*^2^ = 65.69%, Tau^2^ = 0.08; *χ*^2^ = 2.91 (df = 1; *p* = 0.09)	Low	No effect
Pitt et al. (2013) [41, 42]	Satisfaction with service (PSW as an adjunct to care, time point not stated)	2 (125)	Adults with severe mental health diagnoses	SMD, Z	0.76 (− 0.59, 2.10), *Z* = 1.1 (*p* = 0.27)	*I*^2^ = 63.11%, Tau^2^ = 0.64; *χ*^2^ = 2.71 (df = 1, *p* = 0.1)	Low	No effect
Wang et al. (2022) [44]	Satisfaction (last follow-up)	3 (838)	Adult individuals or family members with SMI	SMD	–2.17 (–5.18, 0.84); *Z* = 1.41, *p* = 0.16	*I*^2^ = 100%; Tau^2^ = 7.04; *χ*^2^ = 464.22 (df = 2, *p* < .00001)	Critically low	No effect
Satisfaction with care: follow-up								
White et al. (2020) [45]^a^	Satisfaction with services (12–18 months follow-up)	2 (286)	Adults using mental health services with any diagnoses	SMD, Z	0.19 (− 0.05, 0.42), *Z* = 1.6 (*p* = 0.116)	*I*^2^ = 0%, *χ*^2^ = 0.0 (*p* = 0.878)	Critically low	No effect
Relational support								
Pitt et al. (2013) [41, 42]	Relationship between treatment provider and service user (participant assessed, time point not stated)	2 (160)	Adults with severe mental health diagnoses	SMD	0.22 (− 0.10, 0.53), *p* = 0.18)	*I*^2^ = 0%	Low	No effect
Wang et al. (2022) [44]	Social support (last follow-up)	4 (600)	Adult individuals or family members with SMI	SMD	0.09 (–0.08, 0.26); *Z* = 1.08, *p* = 0.28	*I*^2^ = 0%; Tau^2^ = 0; *χ*^2^ = 2.59 (df = 3, *p* = 0.46)	Critically low	No effect
Wang et al. (2022) [44]	Building positive relationships (last follow-up)	2 (293)	Adult individuals or family members with SMI	SMD	–0.62 (–3.02, 1.78); *Z* = 0.51, *p* = 0.61	*I*^2^ = 99%; Tau^2^ = 2.97; *χ*^2^ = 82.45 (df = 1, *p* < .00001)	Critically low	No effect
Wang et al. (2022) [44]	Family burden (last follow-up)	2 (540)	Adult individuals or family members with SMI	SMD, Z	–1.75 (–3.63, 0.12), *Z* = 1.83, *p* = 0.07	*I*^2^ = 99%, Tau^2^ = 2.69, *χ*^2^ = 143.20 (df = 2, *p* < 0.00001)	Critically low	No effect
White et al. (2020) [45]^a^	Social network support (12–24 months follow-up)	4 (512)	Adults using mental health services with any diagnoses	SMD, Z	0.09 (− 0.25, 0.42), *Z* = 0.5 (*p* = 0.602)	*I*^2^ = 67%, *χ*^2^ = 9.2, *p* = 0.027	Critically low	No effect
Self-efficacy								
Burke et al. (2019) [51]	Self-efficacy (group interventions, end of treatment)	6 (1388)	Adults (including veterans) with any mental health problem	Hedges’ g	0.20 (0.09, 0.31), *p* < 0.01	*I*^2^ = 0%, Tau^2^ < 0.01, Q = 3.55, *p* = 0.62	Critically low	**Significant improvement in self-efficacy at the end of treatment**
Sun et al. (2022) [43]^b^	Self-efficacy (post-intervention)	3 (250)	Adults and adolescents with any mental health problem including MH service users without reported diagnoses	SMD	0.31 (0.06, 0.56), *p* = 0.01	*I*^2^ = 0%, *χ*^2^ = 0.18 (df = 2, *p* = 0.91)	Critically low	**Significant improvement in self-efficacy**
Wang et al. (2022) [44]	Self-efficacy (last follow-up)	3 (301)	Adult individuals or family members with SMI	SMD	0.34 (0.12, 0.57); *Z* = 2.95, *p* = 0.003	*I*^2^ = 0%; Tau^2^ = 0; *χ*^2^ = 2 (df = 2, *p* = 0.37)	Critically low	**Significant improvement in self-efficacy**
Functioning								
Wang et al. (2022) [44]	Psychosocial functioning (last follow-up)	3 (407)	Adult individuals or family members with SMI	SMD, Z	− 2.47 (− 2.95, − 1.98), *Z* = 9.96, *p* < 0.00001	*I*^2^ = 68%, Tau^2^ = 0.12; *χ*^2^ = 6.15 (df = 2, *p* = 0.05)	Critically low	**Significant improvement in psychosocial functioning**
Wang et al. (2022) [44]	Functioning (last follow-up)	7 (1081)	Adult individuals or family members with SMI	SMD	− 0.33 (− 1.44, 0.77); *Z* = 0.59, *p* = 0.55	*I*^2^ = 98%; Tau^2^ = 2.17; *χ*^2^ = 346.35 (df = 6, *p* < .00001)	Critically low	No effect
Wang et al. (2022) [44]	Family functioning (last follow-up)	4 (646)	Adult individuals or family members with SMI	SMD, Z	0.9 (− 0.50, 2.30), *Z* = 1.26; *p* = 0.21	*I*^2^ = 98%, Tau^2^ = 1.94; *χ*^2^ = 160.98 (df = 3, *p* < 0.00001)	Critically low	No effect
White et al. (2020) [45]^a^	General and social functioning (6–12 months follow-up)	3 (181)	Adults using mental health services with any diagnoses	SMD, Z	0.01 (− 0.32, 0.35), *Z* = 0.1 (*p* = 0.937)	*I*^2^ = 21%, *χ*^2^ = 2.5 (*p* = 0.283)	Critically low	No effect
Other psychosocial outcomes								
Sun et al. (2022) [43]^b^	Self-stigma (post-intervention)	7 (580)	Adults and adolescents with any mental health problem including MH service users without reported diagnoses	SMD	− 0.32 (− 0.49, − 0.16), *p* = 0.0001	*I*^2^ = 19%, *χ*^2^ = 7.39 (df = 6; *p* = 0.29)	Critically low	**Significant decrease in self-stigma**
Sun et al. (2022) [43]^b^	Stigma-related stress (post-intervention)	3 (238)	Adults and adolescents with any mental health problem including MH service users without reported diagnoses	SMD	− 0.71 (− 1.11, − 0.30), *p* = 0.0007	*I*^2^ = 58%, Tau^2^ = 0.07, *χ*^2^ = 4.71 (df = 2, *p* = 0.09)	Critically low	**Significant decrease in stigma-related stress**
Fuhr et al. (2014) [37]	Loneliness (post-intervention-12 months follow-up)	2 (641)	Adults service users with an SMI or depression diagnosis	SMD, Z	0.27 (− 0.19, 0.72), *Z* = 1.14 (*p* = 0.25)	*I*^2^ = 57%, Tau^2^ = 0.07; *χ*^2^ = 2.30 (df = 1, *p* = 0.13)	Low	No effect
Wang et al. (2022) [44]	Self-esteem (last follow-up)	3 (759)	Adult individuals or family members with SMI	SMD	− 0.91 (− 2.78, 0.96); *Z* = 0.95, *p* = 0.34	*I*^2^ = 99%; Tau^2^ = 2.71; *χ*^2^ = 223.44 (df = 2, *p* < .00001)	Critically low	No effect

^a^Review included studies of individual peer support only

^b^Review included studies of group peer support only; no stars = Review included studies of either individual or group peer support, or both

### Results from meta-analyses

#### Clinical outcomes

For depression outcomes, evidence from two reviews with meta-analyses suggested that peer support is effective in improving perinatal depression [47, 48]. Three reviews of peer support for adults and adolescents with mental health problems including those with SMI diagnoses reported no effect on depression post-intervention [22, 35, 43], where two of these reviews looked at group-based peer support alone [35, 43]. Two of these reviews reported follow-up results; one review of group peer support for adults with any mental health condition continued to find no effect at 3–6 months follow-up [35], while the other involving adults with SMI reported improvements in depression and anxiety at 6 months follow-up, despite reporting no effect at post-intervention [22]. One review [52] measured clinical recovery in adults with any mental health diagnosis, reporting improvements post-intervention and at 6–9-month follow-up, but no improvement at 12–18-month follow-up.

Most evidence regarding mental health symptom severity among adults and adolescents with mental health diagnoses or who were using mental health services suggested no effect [22, 35, 41–44], other than for perinatal depression as previously summarised. One review [40] of individual peer support for adults with primarily SMI diagnoses reported improvements in symptom severity, while another involving adults with SMI [44] reported symptom improvements following family-led peer support, but no improvement following individual-led peer support. Results for service use varied depending on the measure, for example, peer support was associated with reduced risk of hospitalisation [44], including after a follow-up period [45], but no effect was found regarding length of stay [41, 42].

All reviews providing meta-analytic evidence relevant to this question were rated low or critically low quality, except from one high-quality review [21] which found no effect of peer support on patient activation between 1 and 6 months follow-up (a person’s perceived ability to manage their illness and their approach to healthcare) in adults with schizophrenia diagnoses or similar SMI.

#### Recovery outcomes

Of the seven reviews with meta-analyses reporting data on overall self-reported recovery, five reported improvements in recovery in adults with mental health diagnoses including SMI [22, 35, 40, 44, 45]. Two studies found effects for individual peer support interventions alone [40, 45], and one reported an effect for group-based peer support alone [35]. Only two reviews reported no effect [21, 43], where one included studies of adults with SMI in both individual and group-based peer support [21], and the other involved studies with adults and adolescents with any mental health problem in group-based peer support alone [43].

Three reviews reported follow-up data showing continued improvements for adults with mental health diagnoses including SMI at follow-ups of 6 months [22], 3–6 months [35] and 12–18 months [45], the former and the latter reviewing individual and group peer support, and the second focussing on group peer support alone. One further review reported no improvements at medium-term follow-up (1–6 months) [21]. One review of adults with any mental health diagnosis identified improvements in personal recovery post-intervention, but not at 6–9 or 12–18 months follow-up, and found no improvements in functional recovery post-intervention or at 12–18 months follow-up, but did report improvements at 6–9 months follow-up [52].

All reviews providing meta-analytic evidence for these outcomes were rated as critically low or low quality, except for one [21] which was rated high quality. Based on evidence from three studies, this latter review [21] found no effect of peer support on recovery in the medium term for adults with schizophrenia diagnoses or similar SMI.

#### Psychosocial outcomes

Evidence regarding hope or hopefulness was mixed. Four reviews with meta-analyses suggested that peer support resulted in improvements in adults with SMI [22, 37, 40, 44], where one of these studies looked at individual peer support alone [40] and the rest included both individual and group peer support. However, three reviews of studies including SMI and mixed mental health diagnoses samples reported no effect [21, 35, 43], where two of these reviews focussed on group-based peer support alone [35, 43]. One study [22] followed up adults with SMI and those using secondary MH services at 3–6 months and found continued improvements in hope. However, another review investigating longer-term outcomes (over 6 months) in adults with SMI found no effect [21].

Improvements in empowerment were evidenced by two reviews with meta-analyses [40, 51] of studies involving adults with any mental health diagnosis including SMI. No effects were reported in four reviews [22, 35, 43, 44]. One of the meta-analyses finding positive effects of peer support on empowerment looked at individual peer support alone [40], whereas two of the meta-analyses with no effect solely involved group-based peer support [35, 43]. Three studies reported follow-up data. Two showed improvements at 6 months in adults with SMI [22] and at 6–12 months follow-up among adults using mental health services with any diagnoses [45]. The other showed no improvements from group-based peer support only in adults with mental health diagnoses including SMI between 3 weeks and 6 months follow-up [35].

Quality of life reportedly improved in two reviews with meta-analyses [37, 44] of studies involving adults with SMI, while there was no evidence of improvement in one other with an SMI sample [22]. The two studies which reported follow-up data continued to find no effect [22, 45].

There were improvements in self-efficacy in adults with any mental health problem in all three reviews with meta-analyses reporting this outcome [43, 44, 51]. Decreases in self-stigma and stigma-related stress in adults and adolescents with any mental health problem were found by one review with meta-analysis of group-based peer support [43]. There was no evidence for peer support improving satisfaction with care [22, 41, 42, 44, 45] or relational outcomes (including social support and network) and building relationships (both personally and with staff) [41, 42, 44, 45].

All reviews providing meta-analytic evidence for these outcomes were rated as critically low or low quality, except one high-quality review [21] which found no effect of peer support on hope in adults with schizophrenia diagnoses or similar SMI in the medium or long term.

### Summary of results from systematic reviews without meta-analysis

Effectiveness results from systematic reviews without meta-analyses are tabulated in full in Additional file 1: Appendix 6. These reviews presented mixed results pertaining to clinical outcomes including depression, anxiety, eating disorder pathology, and psychosis. However, two scoping reviews reported evidence of peer support in improving suicidal ideation [57, 58]. Evidence was deemed inconclusive regarding the impact of peer support on indicators of service use, where three reviews failed to find evidence for peer support [21, 22, 41, 42], three reported mixed results [1, 38, 54], and one found evidence for improvements associated with peer support [36]. More consistent evidence was found indicating peer support improves recovery outcomes [1, 36, 38, 40, 44, 53]. For most psychosocial outcomes, systematic reviews presented mixed evidence, for example different effects were found by one high-quality review for empowerment, hope and self-efficacy, depending on what measures were used [21]. Despite mixed effects being reported overall for the impact of peer support on satisfaction with care, one review cited some possible associated moderating factors such as the number of conversations had between peer supporter and recipient [48]. Evidence was marginally less mixed for relational outcomes, such as strength of interpersonal relationships and sense of community, as the majority (three) of relevant reviews found evidence in support of peer support [21, 38, 58], although one review found this did not persist long term [21].

### RQ 2: What influences the implementation of peer support approaches for mental health?

Implementation was investigated in nine reviews [23, 24, 36, 39, 46, 50, 55, 59, 62]. Table 5 shows an overview of implementation outcomes by CFIR domain [33]. All reviews relevant to this research question were rated as critically low quality based on the adapted AMSTAR 2 rating scale (see Additional file 1: Appendix 3).

**Table 5:** Implementation outcomes by CFIR (Consolidated Framework for Implementation Research) domain

Domain	Synthesised data	Reference
**Innovation** *The ‘thing’ being implemented, e.g. a new clinical treatment, educational programme, or city service*	- High acceptability and feasibility of PSW-led support. - Engaging the community in a co-production approach should be adopted in the design of the peer provision service.	[36, 39, 46, 50, 59]
**Outer Setting** *The setting in which the Inner Setting exists, e.g. hospital system, school district, state. There may be multiple Outer Settings and/or multiple levels within the Outer Setting (e.g. community, system, state)*	- Integration of intervention implementation within existing healthcare systems. - National policy initiatives and funding provisions for employing and retaining PSWs. - PSWs having access to a wider peer network. - Interference of work with social security benefits. - Power hierarchies in certain broader cultural contexts. - Difficulties incorporating PSWs in a medical model of mental health care. - A lack of recognised certification for peer workers.	[24, 39, 46, 50, 62]
**Inner Setting** *The setting in which the innovation is implemented, e.g. hospital, school, city. There may be multiple Inner Settings and/or multiple levels within the Inner Setting, e.g. unit, classroom, team*	- Strong leadership and support from leadership at the highest level. - Importance of a workplace culture emphasising recovery-orientated practice. - Employers being flexible and understanding of needs of PSWs. - A supportive, accepting and trusting workplace culture where PSWs occupy a central position within service network and fit in well with other staff members. - Trusting culture allows management of risk in a psychologically safe space. - Access to necessary resources, e.g. desk space, computer, administrative data and medical records. - Time pressure and high caseloads leading to not enough time with patients. - Not enough funding for PSW role and no or limited renumeration for PSWs. - Effective communication and collaboration between PSWs and other workers. - Organisational openness and readiness to employ PSWs. - Organisations encouraging a ‘keeping well at workplan’ to support their PSWs, especially in times of crisis.	[23, 24, 39, 46, 50, 59, 62]
**Individuals** *The roles and characteristics of individuals*	- Professionalisation and legitimisation of PSW role with performance standards/code of ethics. - The use of rigorous recruitment practices to hire PSWs. - High levels of competency among peer-counsellors when delivering interventions and having relevant skills and knowledge, e.g. mental health conditions. - Conflicted sense of identity when constructing either ‘professional identity’ or ‘peer worker identity’. - Required recovery status for peer supporters. - PSWs ability to use coping skills and be resilient to avoid potential negative impacts on their wellbeing. - Staff willingness and ability to work with PSWs and accepting them as part of the service. - The use of champions and implementation leaders to drive the set up and maintenance of PSW interventions. - The use of appropriate confidentiality considerations (e.g. removing PSWs details from the service if they had previously been a patient there).	[24, 39, 46, 50, 55, 62]
**Implementation Process** *The activities and strategies used to implement the innovation*	- Comprehensive training for PSWs delivered prior to starting work and on an ongoing basis. - Training should include practical skills for the PSW role, knowledge and awareness of mental health conditions. - Training other members of staff to effectively work with PSWs. - Regular clinical supervision for PSWs. - Clear role definition for PSW with appropriate boundaries. - Safeguarding precautions, e.g. removal of triggering content; psychiatric assessment and monitoring for PSWs. - Establishing sustainable systems of implementation (e.g. models of cost and supervision) from the outset of the implementation process to sustain PSW engagement over time. - Taking service user and PSW preferences into account when matching based on certain characteristics (e.g. demographics/diagnosis).	[24, 36, 39, 46, 50, 55, 59, 62]

#### Innovation

Studies reported generally high acceptability and feasibility of PSW-led interventions [36, 39, 46, 50]. When planning a peer-led service, co-producing the design of peer support provision with the community and stakeholders was found to be key [59].

#### Outer setting

The existence of national policy and funding provisions for employing and retaining PSWs facilitated PSW-led care [39, 46, 59], as did integration of interventions within existing healthcare systems [50]. However, barriers included power hierarchies [39], difficulties incorporating PSWs in medical mental health care models [24, 39, 46], interference of work with welfare benefits [62] and a lack of recognised PSW certification [62].

#### Inner setting

A workplace culture emphasising recovery-orientated practice [24, 59], and organisational openness and readiness to employ PSWs [39], was important. Facilitators included strong leadership and support at the highest level [46], and flexible and understanding employers, especially in times of crisis [59]. A key facilitator was a supportive, accepting and trusting workplace culture where PSWs occupy a central position and fit in well with other staff members [24]. A trusting culture allowed the management of risk in a psychologically safe space [59]; effective communication and collaboration between PSWs and other workers facilitated this [24], while stigmatising staff attitudes were a barrier [62]. It was easier to implement PSWs in a more collaborative and less hierarchical service [59]. There were practical facilitators and barriers for PSWs also, such as access to desk space or administrative data [24, 46], time restraints, high caseloads [23, 24] and insufficient funding for PSW role [24, 50].

#### Individuals

The professionalisation and legitimisation of the PSW role was seen as important, with associated performance standards and/or a code of ethics [24] which was linked to rigorous recruitment practices, ensuring parity in the recruitment of PSWs and other staff [46]. A further facilitator was high levels of competency among peer-counsellors when delivering interventions and having relevant skills and knowledge, e.g. mental health conditions [50]. PSWs were often required to have recovered from their mental health difficulties [55] and be able to use their coping skills and resilience to avoid potential negative impacts on their wellbeing [24]. PSWs reported a conflicted sense of identity between being a ‘peer’ with experience of mental health problems and a ‘professional’ as a barrier to their work [62]. The use of champions and implementation leaders to drive the set up and maintenance of PSW interventions was reported as a facilitator [46], as was staff willingness and ability to work with PSWs and accept them as part of the service [24].

#### Implementation process

Studies emphasised the importance of comprehensive training for PSWs delivered both prior to starting work and on an ongoing basis, alongside regular clinical supervision [24, 46, 50, 55] supporting the management of any problems encountered [59]. PSW roles should be clearly defined [24, 62] and training should also be delivered to other members of staff to help them work effectively with PSWs [46]. Establishing sustainable models of cost and supervision from the outset was key for the longevity of PSW [50].

### RQ 3: What are the experiences of peer support approaches for mental health (e.g. of acceptability) from the perspective of PSWs, healthcare practitioners, service users and carers?

Experiences of both the benefits and challenges of peer support were reported in 11 reviews [23, 34, 39, 42, 46, 49, 55, 60, 61, 63, 64] from a range of perspectives: PSWs [23, 34, 39, 55, 61], service users [39, 55, 61], non-peer staff [61], peer support group members [49], and mixed samples which consisted of combinations of PSWs, service users, non-peer staff, carers, mental health organisations, policy makers and peer programme developers [23, 39, 42, 46, 55, 60, 61, 63, 64]. In one review, it was unclear whose perspective was being presented [46], although this review only contributed to one theme. All reviews providing evidence for this research question were rated as critically low quality based on the adapted AMSTAR 2 rating scale (see Additional file 1: Appendix 3). We identified 3 overarching themes: (i) what the PSW role can bring, (ii) confusion over the PSW role and (iii) organisational challenges and impact. Table 6 gives an overview of the overarching themes and subthemes (with more detail in Additional file 1: Appendix 7). The following provides an overview of each overarching theme from the perspective of the different samples (i.e. PSWs, service users, mixed samples).

**Table 6:** Experiences of peer support (overview of themes)

Theme	Benefit/ challenge, references	Summary and sample
**What the PSW role can bring**		
**Wellbeing and recovery**	Benefit [23, 34, 61]	**PSWs** [23, 34, 61]: PSWs experienced improved wellness and recovery. The role enabled them to reframe and accept their illness and kept them engaged in recovery. They also experienced increased confidence, social networks, self-esteem, self-knowledge, and personal growth, through, e.g. using their lived experience to help others, a sense of belonging, learning more about their own mental health and learning from service users.
	Challenge [23, 42, 55, 60]	**PSWs** [23, 34]: the role could have a negative impact on PSW wellbeing and recovery, e.g. due to a heavy workload, the role could remind them of their illness and the ‘sick’ label could stay with PSWs. Service users could be a source of stress, e.g. service users who had a greater level of disturbance than the PSWs own experience. **Mixed**^**a**^ [42, 55, 60]: PSW absenteeism due to illness or relapse increased caseload for non-peer staff. There is a risk that service users and PSWs could experience distress due to exposure to triggering content. There was fear that PSWs recovery process could negatively impact the support provided. (service users, PSWs, carers, non-peer staff).
**Recovery and role models**	Benefit [23, 34, 39, 49, 55, 60, 61, 64]	**PSWs** [23, 34, 55]: PSWs felt mutual benefits from the role. The role aided PSWs personal recovery through, e.g. providing a route back into employment and social inclusion. The importance of PSWs being role models was related to embodying personal recovery so they could be ‘the evidence of recovery’. **Service users** [39, 55, 61]: For service users, PSWs could be role models and give service users hope of recovery, e.g. from working with PSWs, service users experienced increased hope, motivation, better social communication skills, a sense of belonging and improved mental health symptoms. PSWs could show service users that life beyond illness is possible. Service users valued PSWs sharing their knowledge and felt empowered as they gained knowledge on mental health. Gaining knowledge motivated service users to be optimistic and independent in their recovery. **Non-peer staff** [61]: From working with PSWs, non-peer staff developed increased empathy towards people in recovery and a belief in recovery. **Peer support group members** [49]: Forming relationships in peer support groups was valuable for recovery, e.g. enabled re-evaluation of self and expectations [of motherhood]. **Mixed**^**a**^ [60, 64]: PSWs are role models, give service users hope of recovery, are valued and provide guidance and support to service users through the process of engaging with mental health services, e.g. how to navigate services. (non-peer staff, PSWs, service users, policy makers, peer programme developers, carers)
	Challenge [61]	**Service users** [61]**:** Some reported that PSWs are not role models for service users. Reasons included a belief that without formal training and because of their mental health diagnosis PSWs would be ineffective helpers.
**Career, social inclusion and identity**	Benefit [23, 34, 42, 61]	**PSWs** [23, 34, 61]: The PSW role enabled them to contribute through work, which helped maintain recovery. The role offered a route back into employment, gaining skills, financial freedom, structure and stability, improving functioning and increasing social inclusion (e.g. by interacting with non-peer staff, on an equal footing), and social networks PSWs reported increased self-acceptance as they no longer had to hide their mental health issues. The role could also be a stepping stone into further employment. **Mixed**^**a**^ [42, 61]: PSW roles were rewarding and enabled service users to find a place in the community beyond ‘patient’. (Mental health organisations, PSWs, non-peer staff, service users, carers)
**Experiential knowledge, normalisation and stigma**	Benefit [39, 55, 61]	**Service users** [39, 55]**:** For service users, PSW support differed from formal treatment, it normalised and de-medicalised service user experiences. This difference felt person-centred leading service users to reconnect with ‘real life’ situations, e.g. rebuilding relationships. Lack of judgement from PSWs reduced stigma around service users’ experiences of an eating disorder. The sense of a ‘shared experience’ helped service users feel they were ‘getting back to normal’. Service users valued peer support services and appreciated PSWs experiential knowledge, perceiving them to be more insightful than non-peer staff as they were viewed as role models in recovery, promoting empowerment and hope for service users. PSW services were trusted, making service users feel comfortable and accepted when attending activities. **Mental health organisations** [61]**:** For organisations, PSW roles decreased mental health stigma and set a positive example to other sectors
	Challenge [39]	**Service users** [39]: Some service users and members of the public found it challenging to view PSWs as mental health professionals due to concerns on their mental health history. Some service users perceived the knowledge of PSWs to be of lower value than that by healthcare professionals and should not be fully trusted.
**Isolation and validation**	Benefit [49]	**Peer support group members** [49]: Having their experiences, e.g. that mothering in illness is difficult, validated by other mothers made life ‘less difficult’.
	Challenge [49]	**Peer support group members** [49]: Meeting other mothers could lead to increased isolation, where their experiences were contrasting, e.g. feeling that others are happy when they are not.
**Rapport and empathy with service users**	Benefit [39, 61]	**Service users** [39, 61]: Service users built rapport easier with PSW than non-peer staff due to PSWs having less professional distance and being ‘street smart’. Service users felt that PSWs were more approachable and caring than non-peer staff, enabling them to open up and share concerns. Service users perceived greater empathy from PSWs, especially regarding adverse effects from medications.
**Bridge**	Benefit [64]	**Mixed**^**a**^ [64]: PSWs function as a bridge between service users and non-peer staff and within the organisation, by building trust-based pathways, supporting the service user across the fragmented care system. (non-peer staff, PSWs, service users, policy makers, peer programme developers)
**Pioneer and expectations**	Challenge [64]	**Mixed**^**a**^ [64]: PSWs were pioneers which led to expectations and pressure, i.e. no room for failure which would reduce future PSW opportunities. (non-peer staff, PSWs, service users, policy makers, peer programme developers)
**Complementary role, expertise and becoming part of the team**	Benefit [64]	**Mixed**^**a**^ [64]: Non-peer staff recognised the valuable contribution of PSWs and PSWs fit with various perspectives, becoming a team member. E.g. they provided psychosocial support, were sources of experiences, fresh insights, and information, and had time to do tasks that others may not, e.g. time to just talk to patients. Collaborating with PSWs could improve recovery-oriented care. PSWs may acquire different knowledge about service users than non-peer staff, e.g. about drug abuse. (non-peer staff, PSWs, service users, policy makers, peer programme developers)
	Challenge [64]	**Mixed**^**a**^ [64]: PSWs may lack a broader perspective on mental health beyond their own experience. (non-peer staff, PSWs, service users, policy makers, peer programme developers)
**Confusion over the PSW role**		
**Role ambiguity**	Benefit [64]	**Mixed**^**a**^ [64]: When PSWs were introduced, their role was ambiguous. This was positive as it gave flexibility to define the role (non-peer staff, PSWs, service users, policy makers, peer programme developers)
	Challenge [32, 38, 42, 43, 53, 54]	**PSWs** [23, 34]**:** A lack of clarity about the PSW job description meant that PSWs felt confused in their role which affected their confidence, perception of competence, with ramifications for their recovery and uncertainty in their responsibilities to service users. A lack of clarity also led PSWs to feel the role was tokenistic, and to feel uncertain about where to seek support. **Service users** [39, 55]**:** Some service users perceived a lack of clarity on the PSWs' roles: PSWs were viewed as informal staff who were replaceable, leading to negative perceptions of the PSW services. Some service users perceived peer support to be tokenistic, which led to the content of the PSW intervention ‘feeling irrelevant’. **Mixed**^**a**^ [42, 63, 64]**:** PSWs found their role ambiguous making them anxious to demonstrate their value. PSWs felt they received insufficient training and were expected to develop the role over time, this hampered service delivery, creating the perception that PSWs were tokenistic. Non-peer staff were unsure of the PSW role, leading to a lack of support from non-peer staff. (PSW, non-peer staff, service users, carers, policy makers, peer programme developers).
**Disclosure of peer status**	Challenge [34, 39, 63, 64]	**PSWs** [34, 39]**:** PSWs differed in how comfortable they felt in disclosing their recovery story. For some PSWs sharing their story was connected to their personal recovery. Some PSWs expressed fears of being socially excluded and labelled as ‘mentally ill’ thus would avoid sharing their experiences because they believed service users would not trust them or value their knowledge. PSWs also expressed concern about getting jobs outside of mental health due to their peer worker identity. **Mixed**^**a**^ [63, 64]: There was confusion over when/with whom to disclose lived experience. For example, disclosure was important to educate team on alternative views but may require discretion within professional relationships. But ‘professionalism’ may not challenge existing boundaries which could change culture. Some PSWs felt vulnerable and were reluctant to disclose but disclosure could build trust with service users and enabled PSWs to be recovery role models. (PSW, service users, policy makers, peer programme developers, non-peer staff, mental health organisations).
**Boundaries**	Challenge [23, 60, 61, 63]	**PSWs** [23, 61]**:** the transition from service user to PSW and knowing where to draw the line between friend and service provider, was challenging. Working as a PSW in substance abuse could lead to disconnection from their own recovery communities due to ethical concerns when sharing in support groups, putting the PSWs recovery at risk. **Mixed**^**a**^ [60, 63]**:** whether PSWs should relate to service users as friends (seen as unprofessional) or service users. Some PSWs would not share service user information with agency staff due to concern about violating friendship. (Service users, PSWs, carers, non-peer staff)
**Role conflict and professionalization**	Challenge [34, 61, 63, 64]	**PSWs** [34]**:** for PSWs dual identity as a service user and service provider could be a source of stress and impact on relationships and boundaries. For example, PSWs could more closely connect with service users with similar difficulties to their own but this could have an emotional impact and could be triggering for PSWs leading to a recurrence of their own mental health issues. PSWs found the dual identity particularly difficult where PSWs were working in a team that previously cared for them. **Mixed**^**a**^ [61, 63, 64]: The transition from patient to staff is challenging. For example, non-peer staff may be concerned about the PSW becoming unwell, especially if they were previously a patient at the facility, making PSWs feel that they’re being treated like patients. PSWs can be ‘unwilling’ to give up their consumer perspective to adopt ‘professional beliefs and roles’, e.g. training was questioned as leading to professionalisation and interference with the advantage of being a PSW. (PSW, service users, policy makers, peer programme developers, non-peer staff, mental health organisations).
**Organisational challenges and impact**		
**Lack of support and training**	Challenge [23, 34, 60, 64]	**PSWs** [23, 34]: PSWs experienced a lack of support and training, potentially related to unclear job descriptions. PSWs struggled to develop the skills for their roles, including to work with service users with more complex needs than their own experiences. PSWs reported their supervision felt superficial, and problems in their relationship with their supervisors, e.g. due to PSWs not feeling that they had enough autonomy. **Mixed**^**a**^ [23, 60, 64]: It was felt that lived experience wasn’t solely sufficient to work in interprofessional teams. Some PSWs were positive about certification, others felt that certification could conflict with the grassroots, user-led ethos. Supervision and support were often not offered to PSWs. Risks might arise due to PSWs lack of training and support. Organisations needed to train PSWs and non-peer staff about the value of peer support and develop/implement guidelines. (PSW, non-peer staff, service users, carers, policy makers, peer programme developers).
**The value of the PSW role and low pay**	Challenge [23, 34, 61, 63, 64]	**PSWs** [23, 34, 61]**:** The value of the PSW role was linked to low pay. There were concerns about low pay, few hours and working overtime without compensation. Low pay contributed to role dissatisfaction with PSWs viewing themselves as ‘cheap labour’. However, some PSWs felt that they were well compensated. **Mixed**^**a**^ [63, 64]**:** PSWs received low pay. This was difficult as they wanted jobs that freed them from disability income. Low pay contributed to role dissatisfaction and suggested the job was new, not valued or unclear. PSWs felt pay correlated with legitimacy and tokenism. Reasons for low pay were hourly pay, PSW not requiring certification, stigma from non-peer staff about 'the capacity for people with mental health conditions to work'. (non-peer staff, PSWs, service users, policy makers, peer programme developers)
**Workload**	Challenge [64]	**Mixed**^**a**^ [64]: PSW workload could be overwhelming. This could jeopardise other staff relationships, also under pressure from their own workload. Being given so many varying tasks (e.g. household tasks, meetings) the role could lose its distinctiveness. This was added to by a lack of understanding of the PSW role. (non-peer staff, PSWs, service users, policy makers, peer programme developers)
**Colleagues and stigma**	Challenge [23, 34, 39, 46, 61, 64]	**PSWs** [23, 34, 61]: Although PSWs reported feeling accepted in their teams, some PSWs could experience negative and rejecting non-peer staff attitudes, e.g. treated as a patient, rather than a colleague, talking inappropriately or joking about people with mental health issues, PSWs not invited to social events. PSWs felt excluded, experienced tokenism and stigma, this could lead to isolation and self-stigma. **Non-peer staff** [61]: There was fear that ‘cheap labour’ provided by PSWs might lead to less non-peer staff positions. **Mixed**^**a**^ [39, 46, 64]: PSW roles could be a threat to other professionals’ roles, e.g. nurses suspicious they may be replaced. Non-peer staff were uneasy about working with people they had previously treated or PSWs seeing medical records, e.g. of other PSWs. Concerns from healthcare professionals and policymakers over effectiveness and safety of peer support led to a lack of support and hostility from non-peer staff. Hence PSWs were accorded less respect and fewer responsibilities, with doubts consequently cast over their credibility. PSWs felt uncomfortable talking about their role due to stigma, they challenged stigma by taking on more responsibility. Hierarchies in teams undermined PSWs feeling equal in meetings, they needed to find their voice to challenge clinically dominant ways of thinking. (PSW, service users, policy makers, peer programme developers, non-peer staff, mental health organisations, unspecified (in one study)).
**Challenges for healthcare staff/organisations**	Challenge [42, 61]	**Mixed**^**a**^ [42, 61]: Non-peer staff felt there were expectations to support, train and supervise PSWs, increasing their workload. Some staff found it challenging to have different ‘providers’ [PSWs] in the team. Confidentiality, disclosure and increased sick time of PSWs compared to non-peer workers were issues for organisations. (Service users, PSWs, carers, non-peer staff, mental health organisations).
**Treatment models**	Challenge [23]	**PSWs** [23]: PSWs are part of the newer recovery model and had trouble integrating into the traditional treatment model, e.g. where doctors held majority of power and decision making for service users but spent the least time with service users. PSWs were expected to contest the traditional treatment model in support of a recovery focus (e.g. by their presence or in some cases by being openly challenging), this led to friction. If organisations are not prepared for PSWs the role doesn’t provide stable employment.
**Other**		
**Offering treatment choice**	[60]	**Mixed**^**a**^ [60]: Service users should have opportunities to choose among PSWs as service providers. (service users, PSWs, carers, non-peer staff).

*PSW* Peer support worker

^a^For ‘mixed’ samples the specific sample that stated the theme is unknown (e.g. PSW or non-peer staff or both)

### What the PSW role can bring

#### Perspective of PSWs

PSWs experienced improved wellness and recovery from working in the role, reporting increased self-esteem, personal growth, and social networks [23, 34, 55, 61]. They benefited in a variety of ways, e.g. the role provided a route back into employment, improving functioning and social inclusion, and allowed them to learn more about their own mental health [23, 34]. PSWs also reported increased self-acceptance as they no longer had to hide their mental health issues [34]. The role was therefore often reported to be mutually beneficial for PSWs and service users [34, 55]. PSWs felt it was important that they were role models for service users, being ‘the evidence of recovery’ [34]. However, working as a PSW could also have a negative impact on the PSWs’ wellbeing and recovery [23, 34]. Reasons for this included the role reminding them of their mental health condition and the ‘sick’ label staying with them [23].

#### Perspective of service users

For service users, PSWs could be role models, giving them hope of recovery [39, 55, 61]. PSW support normalised and de-medicalised service user experiences [55]. Lack of judgement from PSWs reduced feelings of self-stigma for service users [55]. Service users felt empowered by and valued gaining experiential knowledge from PSWs, perceiving them to be more insightful than non-peer staff, and trusting their services [39]. Service users also built rapport more easily with PSWs than non-peer staff, feeling they were more approachable and had greater empathy than non-peer staff [39, 61]. However, some service users reported that PSWs are not role models and found it challenging to view them as professionals or fully trust their knowledge, due to their lack of training and concerns about their mental health history [39, 61].

#### Perspective of non-peer staff

From working with PSWs, non-peer staff developed increased empathy towards service users and a belief in recovery [61].

#### Perspective of peer support group members

Forming relationships in peer support groups and having their experiences validated by others was valuable for recovery [49]. However, group members could feel isolated when other members’ experiences contrasted with their own [49].

#### Perspective of mixed samples

PSWs were perceived to be role models, providing valuable support to service users and giving them hope of recovery [60, 64]. Working as a PSW could enable service users to find a role in the community, beyond the identity of being a ‘patient’ [61]. PSWs could build trust-based pathways to function as a bridge between service users and non-peer staff [64]. Within teams, working with PSWs could improve recovery-oriented care and PSWs carried out various roles, such as providing psychosocial support, advocating for service users, providing insights based on their lived experiences [64]. For mental health organisations, PSW roles decreased stigma towards mental health problems and set a positive example [61]. However, there were fears that the PSWs’ mental health condition could impact the provided support, such as increased PSW absenteeism which could increase non-peer staff caseloads and concerns that service users’ and PSWs’ could experience distress due to exposure to difficult (‘triggering’) content [42, 55, 60]. PSWs experienced pressure due to the perception that they were pioneers, leading to expectations, e.g. failure could reduce future PSW opportunities [64]. There was also concern that PSWs lacked mental health knowledge, beyond their own experience [64].

### Confusion over the PSW role

#### Perspective of PSWs

A lack of clarity about the PSW job description led PSWs to feel the role was undervalued and tokenistic and meant they felt confused in their role. This impacted their perception of competence which affected their recovery and led to uncertainty in their responsibilities with service users [23, 34]. PSWs also found the transition from service user to PSW and knowing where to draw the line between friend and service provider to be challenging [23, 61]. Linked to this, their dual identity as a service user and provider could be a source of stress. For example, it meant they could closely connect with service users who had similar difficulties to their own, but this could also be triggering and lead to a recurrence of the PSWs’ own mental health issues [34]. PSWs expressed varying views on disclosing their recovery story [34, 39]. For some, sharing elements of their story was linked to their own personal recovery [34]. However, other PSWs felt fearful of disclosure, e.g. they were concerned about being labelled ‘mentally ill’ and service users not trusting them [39].

#### Perspective of service users

A lack of clarity on the PSW role could lead service users to view the role as informal, leading to negative perceptions of the PSW services. Perceptions of tokenism of peer support could lead to the content of the PSW intervention ‘feeling irrelevant’ [39].

#### Perspective of mixed samples

PSWs and non-peer staff found the PSW role to be ambiguous, e.g. the role was not clearly defined [63] and job descriptions were ‘vague’ [64]. Although this gave flexibility to define the role [64], it also led to challenges. Some PSWs felt they were expected to develop the role over time and received insufficient training, which hampered service delivery and could result in perceptions that PSWs were tokenistic [42, 63, 64]. Uncertainty about the role also led to a lack of support from non-peer staff [63]. Relatedly, there was confusion for PSWs over when/with whom to disclose their lived experience [63, 64]. Some PSWs felt vulnerable and were reluctant to disclose, but disclosure could build trust with service users, enabled PSWs to be recovery role models, and could educate non-peer staff on alternative views [63, 64]. Disclosure was also felt to require discretion when fitting with professional relationships. However, ‘professionalisation’ of PSWs may not challenge the existing boundaries (e.g. traditional hospital-based boundaries which could make it difficult for the sharing of lived experience to be valuable), when challenging these boundaries could change culture [63, 64]. The transition for PSWs from patient to staff was challenging, e.g. non-peer staff were concerned about the PSW becoming unwell, making PSWs feel like they are being treated like patients [63, 64]. There were issues around boundaries, including whether PSWs should relate to service users as friends or service users [63].

### Organisational challenges and impact

#### Perspective of PSWs

PSWs experienced a lack of support and training for their role, potentially related to unclear job descriptions, and insufficient supervision [23, 34]. This meant that PSWs struggled to develop the skills for their roles, including to work with service users with more complex needs than their own experiences [23]. Although there were some contrasting views, PSWs were concerned that they received low pay which made them feel that they were not valued, and they perceived themselves to be ‘cheap labour’ [23, 34, 61]. Some PSWs felt accepted in their teams however others experienced negative and rejecting non-peer staff attitudes [23, 34, 61]. For example, PSWs reported not being invited to social events and being treated like patients [61]. Consequently, some PSWs felt excluded, that their roles were tokenistic and experienced self-stigma [23, 34]. PSWs as part of the newer recovery model reported challenges around integrating into traditional treatment models, e.g. where doctors spent the least time with service users but held the majority of power and decision making for service users. PSWs were expected to contest the traditional treatment model in support of a recovery focus, e.g. by their presence or in some cases being openly challenging, and this clash between old and new treatment models could lead to friction [23].

#### Perspective of non-peer staff

There was a fear that ‘cheap labour’ provided by PSWs may lead to fewer non-peer staff positions [61].

#### Perspective of mixed samples

PSWs often received low pay, which led to role dissatisfaction for PSWs, suggesting the job was tokenistic or the role was unclear [63, 64]. One reason for low pay was due to PSWs not requiring certification (i.e. specific qualifications, which e.g. a social worker would require) [63]. Some PSWs were positive about certification but others felt it could conflict with the grassroots ethos of peer support. However, there was the view that lived experience was not solely sufficient to work in interprofessional teams [64]. Despite this, supervision and support were often not offered to PSWs leading to risks [60, 64].

There were challenges in PSW relationships with non-peer staff which could lead to a lack of support and hostility from non-peer staff. Non-peer staff felt threatened that they may be replaced by PSWs [64], were uneasy about working with people they previously treated [46], were concerned about the effectiveness of peer support [39], and felt expectations to support PSWs, increasing their workload [42]. This undermined the role of PSWs, e.g. they were subsequently given fewer responsibilities [39]. For PSWs, they wanted to challenge stigma by taking on more responsibility but high, varying workloads could jeopardise relationships with non-peer staff and team hierarchies hindered their ability to challenge clinically dominant ways of thinking [64].

### Other

#### Perspective of mixed samples

A final theme was the perception that service users should be able to choose among PSWs as service providers [60].

### Summary of key findings

An overview and summary of the key findings for each research question is presented in Table 7.

**Table 7:** Overview of key findings

Research question	*N* Reviews	Key findings
1. What is the effectiveness (e.g. clinical, social, functional) and cost-effectiveness of paid peer support approaches for mental health?	23 reviews	• Results were mixed. • There was some evidence from meta-analyses that peer support may improve depression symptoms (particularly perinatal depression), self-efficacy, and recovery.
2. What influences the implementation of peer support approaches for mental health?	9 reviews	• Factors promoting successful implementation, included adequate training and supervision, a recovery-oriented workplace, strong leadership and a supportive and trusting workplace culture with effective collaboration. • Barriers included lack of time, resources and funding, and lack of recognised PSW certification.
3. What are the experiences of peer support approaches for mental health (e.g. of acceptability) from the perspective of PSWs, healthcare practitioners, service users, carers?	11 reviews	Three overarching themes: • ‘What the PSW role can bring’, including recovery and improved wellbeing for service users and PSWs. • ‘Confusion over the PSW role’, including role ambiguity and unclear boundaries. • ‘Organisational challenges and impact’, including low pay, negative non-peer staff attitudes, and lack of support and training.

## Discussion

### Key findings

Our umbrella review of 35 reviews explored the effectiveness, implementation and experiences of peer support for mental health.

Effectiveness was reported in 23 reviews. Many reviews reporting effectiveness data reported no effect of peer support on a range of outcomes, mirroring the findings from other reviews [9, 66] including those focusing on other types of peer support (e.g. online peer support for young people) [67]. However, there was consistent evidence from meta-analyses that peer support may improve the clinical outcomes of perinatal depression and risk of hospitalisation of adults with severe mental illness, as well as recovery outcomes, and self-efficacy and stigma-related outcomes. Mixed meta-analytic results were found for the clinical outcomes of overall psychiatric symptoms in adults with SMI, psychosis symptoms, length of hospital stay and patient activation, and for psychosocial outcomes such as hope, empowerment, and quality of life. There was no meta-analytic evidence for improvements in relational support. Evidence from systematic reviews without meta-analysis similarly gave a mixed picture regarding psychosocial and clinical outcomes, but indicated more consistent evidence that peer support has a positive impact on recovery, suicidal ideation, and, to some degree, satisfaction with care.

Many possible sources of heterogeneity across the included reviews could contribute to the mixed findings in this study, such as low-quality methodologies, differences in the populations included, and poor specification of peer support roles or the content of interventions delivered. One important potential contributor to our mixed results is that the primary studies contributing to the included reviews often varied in the type of control groups they considered, for example studies with treatment as usual, active controls and waitlist controls were often reviewed within the same paper. As such, it was not possible to determine whether peer support is effective in comparison to certain types of care provision but not others. In a similar vein, we could not perform subgroup analysis to determine whether specific forms of peer support are more effective on certain populations as most reviews with meta-analyses involved a combination of different formats and a range of participant groups. Nevertheless, there was some indication that differences in the format of peer support may impact its effectiveness on empowerment, as the two meta-analyses involving individual peer support alone found a positive effect on empowerment, but the two looking at group-based peer support alone did not. However, further research is needed to adequately address such questions.

Although this overview of quantitative evidence does not give unequivocal support for peer support on a variety of outcomes, the mixed results must be understood not only in the context of heterogeneity of the quantitative research conducted thus far, but with regard to the qualitative evidence documenting strong support for this intervention (as discussed in more detail below). Given that the implementation of peer support in mental health services is still relatively rare and highly variable, many of the trials conducted thus far may have tested peer support in environments where it is not fully embedded in the organisation and culture. Indeed, peer support may have positive impacts on the operation of mental health services that have not been measured as quantitative outcomes in existing trials—such as a stronger culture of person-centred care. More consistent quantitative results demonstrating the benefit of peer support may increasingly emerge as it becomes better integrated in the mental health care system.

We identified several factors reported to be important for the successful implementation of peer support, which were summarised and structured using the CFIR. These factors included adequate training and supervision for PSWs, a recovery-oriented workplace structure, strong leadership and a supportive and trusting workplace culture with effective collaboration between PSWs and non-peer staff. Barriers to peer support being implemented effectively included a lack of time, resources, and appropriate funding, and a lack of recognised PSW certification. Policy, research and campaign groups have advocated implementation approaches in line with these findings, for example, ImROC (implementing Recovery through Organisational Change) [14, 68], who support peer support implementation globally and international competence frameworks from New Zealand [69, 70], outline recovery focus as a core principle of peer support and emphasise the importance of training and ongoing professional development; peer support practice guidelines in the USA outline the importance of and give guidelines for supervision [71]. Formalised career pathways for PSWs [72] may help to address some of the identified barriers to effective implementation of peer support work, although these are still early in their development [68].

Experiences of peer support were from a range of perspectives (e.g. PSWs, service users, non-peer staff) and were organised under three main themes. The benefits of peer support for PSWs, service users and non-peer staff were expressed in many reviews; however, there were also conflicting and challenging experiences of the role. The mental health experience of PSWs was viewed as valuable, but also subject to some stigmatising views. For PSWs, the role could improve their personal wellness and recovery, providing a route back into employment and improving functioning, and provide service users with role models of recovery. The reciprocal benefits of peer support have also been highlighted as an advantage of peer support in resources developed by NHS England [19]. However, PSWs reported the ‘sick’ label stayed with them in the role, with non-peer staff at times concerned that PSWs mental health would impact their work, and some service users reported that they found it challenging to trust PSWs knowledge due to their lack of training and mental health history. A key experience, which became the core of our second theme, was the ambiguity of the PSW job description, including lack of clarity over boundaries with service users and when to disclose PSWs’ personal experiences. This ambiguity meant that the role was flexible, but also led to the perception that it was tokenistic and left PSWs feeling confused which impacted their own recovery. IMROC recommend the prioritisation of clear roles when implementing peer support [68]. Professional accreditation can counter the view of peer support as tokenistic, e.g. the UK Peer Support Competence Framework outlined by the Royal College of Psychiatrists [73] and the Canadian Peer support Accreditation and Certification, a national standard endorsing peer support work as a valuable career, developed in 2017 by PSWs themselves [74]. The final theme ‘organisational challenges and impact’ included experiences such as PSWs receiving inadequate support, training and supervision, and receiving low pay, leaving them feeling undervalued. Some non-peer staff attitudes were also a reported issue; while some PSWs felt accepted within teams, others experienced negative and rejecting non-peer staff attitudes, such as being treated as patients and not being invited to staff social events. Organisations should prepare, structurally and culturally, for the introduction of PSWs in order to ensure PSW wellbeing and reduce the risk of absences due to sickness [68, 75].

### Strengths and limitations

We conducted a comprehensive search of several relevant databases and identified a large number of reviews for inclusion, providing the first detailed summary of review findings relating to effectiveness, implementation and experiences of peer support. We also had consistent involvement of researchers with lived experience of mental health and peer support delivery and receipt throughout the design, data screening and extraction, analysis and synthesis, and manuscript drafting for this paper, which allowed lived experience priorities and experiences to guide our approaches to data and our decision making throughout.

We aimed to focus our review on paid peer support; however, this information was underreported in the reviews, and even when reported, interventions were often grouped with peer support interventions that did not fully meet our eligibility criteria (e.g. were unpaid). We also synthesised data from studies where payment status of PSWs was ambiguous, i.e. not reported. This limits our ability to draw firm conclusions around paid peer support specifically, as a significant portion of the data synthesised was from studies investigating unpaid or voluntary peer support. Another limitation was the lack of involvement of people with lived experience in the included reviews, with involvement reported in only one review [57]. Given the service user-led origins of peer support, future reviews should ensure involvement of people with lived experience. This is addressed in more detail later in this paper. Most included reviews were appraised by the AMSTAR 2 as low or critically low (97%) quality with only one review appraised as high quality. Although the low quality of reviews is a limitation, we aimed to report an overview of all current evidence for peer support to inform policy makers and healthcare practitioners, therefore to maximise the evidence base, we synthesised the reviews scored as ‘critically low quality’. Our ratings are also in line with a prior umbrella review of peer support which rated 87% of reviews as critically low quality and the remainder as low quality, but reported outcomes from all reviews [66].

Beyond the aforementioned limitations regarding variation in studies within each review, there is also a loss of granular detail through the umbrella review process of summarising data across reviews, which themselves contain many studies which have been summarised. The person-centred nature of peer support may mean that there are meaningful outcomes for the service user which are not easily captured in standard outcome measurement tools or recognised as clinically significant. Variation in peer support roles across studies may have contributed to the contradictions in our findings for RQ3, e.g. the challenges around PSW roles being ambiguous, but also the reported benefits of a flexible role.

A strength of our review was our broad inclusion criteria, for example, for qualitative data on experiences of peer support we reported data from the perspectives of service users, non-peer staff and PSWs. Though some data was reported separately by role, there were studies where experiences were reported together, and these perspectives were difficult to disentangle. Finally, we did not conduct a formal meta-synthesis of the qualitative experiences data; therefore, some detail may have been missed.

### Implications for practice

Peer support may be effective at improving some clinical outcomes, self-efficacy and recovery outcomes for some people and could augment the standard service range. Certain groups may benefit from peer support more than others; evidence was strongest for depression outcomes within perinatal populations, but extremely variable for other populations. Peer support may differ in effectiveness depending on population needs and characteristics. PSWs need adequate pay, clear role descriptions and guidelines (e.g. about boundaries and disclosure), ongoing training and supervision, and opportunities for progression. Attitudes about peer support held by non-peer staff may significantly support or impede the implementation and experience of PSWs, and non-peer staff may require training about PSW roles and how to work collaboratively with PSWs. Culture, hierarchical structure and staff acceptability of peer support impact implementation and experience of peer support—structural and cultural change may be required for peer support to succeed, e.g. ensuring a recovery-oriented care model is operating in the service.

### Implications for policy

Successful implementation of PSWs in healthcare settings is likely to require a coproduction approach with clearly defined PSW roles, a receptive hierarchical structure and staff, strong leadership and appropriate training (for PSWs and staff) with clinical and/or peer supervision alongside safeguarding. Issues relating to cost, lack of time and lack of resources are key considerations for service providers aiming to implement PSW that is sustained and effective within services. Additionally, Services could benefit from clear, coproduced guidelines, outlining the steps that are most likely to lead to successful PSW implementation.

### Implications for research

Future primary and secondary research could usefully explore the differences in efficacy, implementation and experiences in paid PSW over time as it becomes more established; an important distinction as there are likely to be differences in these outcomes as the role of PSW develops. Such studies could consider using more personalised outcome measures such as goal-based outcome measurement [76]. Current PSW roles are still poorly defined and PSW content, including PSW variations (such as whether PSWs should deliver structured or more loosely structured, informal interventions, or whether interventions should vary according to need and context), need further exploration. Realist investigations around what works for whom, how and in which contexts would uncover more fine-grained detail on the specific contexts and mechanisms that explain these differences. Very few reviews included in this umbrella review reported lived experience researcher leadership or involvement in the undertaking of the study. It is imperative for future research in this area to appropriately reflect the priorities of those who are directly involved in PSW, either as providers or as service users. As the number of PSWs increases and more formalised roles are created, positive impact may not be restricted to outcomes of those supported by PSWs, but also to the functioning of services at an organisational level [68]. Further research is needed to evaluate how teams function with and without PSWs in order to understand how they may impact experiences through changes at a system level [68].

## Conclusions

Our umbrella review has summarised data from 35 reviews on the effectiveness, implementation, and experiences of peer support for mental health. Although we attempted to focus solely on paid peer support, this detail was often not reported in the reviews. While data on effectiveness was mixed, there was some evidence of improvements on outcomes including depression, particularly perinatal depression, self-efficacy, and recovery, illustrating the potential benefits of wider PSW implementation across mental health services. Good implementation of peer support depends on co-design with people with lived experience, clear job descriptions, a recovery-oriented workplace culture, strong leadership, appropriate training for PSWs and staff**,** and supervision for PSWs. However due to limited information on cost or cost-effectiveness, we are unable to draw conclusions around resources required to implement PSWs. Experiences of peer support were from a range of perspectives. Peer support was mutually beneficial for PSWs’ and service users’ wellbeing and recovery and PSWs became role models. However, at times PSW roles were ambiguous, this meant that the role was flexible but could also lead to confusion which could impact PSWs own recovery. Potential strategies to successfully implement peer support include that the PSW roles should be clear, PSWs should be appropriately trained and paid, as well as supported and supervised within a trusting and accepting workplace structure and culture that advocates for a recovery-oriented model of care.

## Lived experience commentary, written by LM and KM

This study provides a useful summary of the available research on peer support. By providing an overarching review of 35 reviews including 426 available studies, the paper brings together the knowledge on a topic of growing importance and understanding of the experiences, effectiveness, and implementation of peer support. However, this evidence is limited to ‘paid peer support workers’ included in data from academic literature of systematic reviews.

The nature of an umbrella review means that the systematic reviews themselves are synthesised, limiting our ability to look at specific details in the primary studies, for example to look for evidence of lived experience involvement or co-authorship or demographics of participants. The papers within the review are likely to have originated from traditionally funded research enquiries, and an umbrella review potentially magnifies academic or clinical perspectives over user voices and interests. While this is a frustration in any mental-health-related topic, this is particularly concerning in relation to peer support, with its origins in our user-led history.

The roots in user-led peer support are also overlooked when limiting the studies to paid peer support work. Although they might use the same language of mutuality and reciprocity, the two feel different. We are hesitant to suggest that we would prefer the skills and expertise of our supporters to be voluntary and unpaid; we strongly believe their expertise should be valued and funded. But there is something magical about informal peer support which can be lost when it is over-policed in bureaucratic cultures. Additionally, with studies included in the review dating back to 1979, we question how relevant these studies are in informing England’s evolving peer support landscape.

A crucial area of future research is exploring what type of peer support works best for whom and in what circumstances, and how we can deliver this. Furthermore, we need to better understand how NHS cultures can be supported to value the expertise that originates in our lived experience, including the marginalised experiences which have been disproportionately represented in mental health services.

### Supplementary Information

**Additional file 1: Appendix 1.** Prisma checklist [29]. **Appendix 2.** Full search strategy. **Appendix 3.** AMSTAR2 ratings. **Appendix 4.** Excluded studies following full text screening, with reasons. **Appendix 5.** Study overlap. **Appendix 6.** Effectiveness of peer support outcomes: results for non-meta-analysis results. **Appendix 7.** Experiences of peer support (detailed themes).

## Data Availability

The data used and/or analysed during the current study are available from the corresponding author on reasonable request.

## Abbreviations

AMSTAR 2: A MeaSurement Tool to Assess systematic Reviews
CFIR: Consolidated Framework for Implementation Research
ImROC: Implementing Recovery through Organisational Change
LEWG: Lived Experience Working Group
PICO: Population, Intervention, Comparator group, Outcome
PSW: Peer support worker
